# Super-enhancer associated core regulatory circuits mediate susceptibility to retinoic acid in neuroblastoma cells

**DOI:** 10.3389/fcell.2022.943924

**Published:** 2022-09-06

**Authors:** Roshna Lawrence Gomez, Laura M. Woods, Revathy Ramachandran, Ahmad N. Abou Tayoun, Anna Philpott, Fahad R. Ali

**Affiliations:** ^1^ College of Medicine, Mohammed Bin Rashid University of Medicine and Health Sciences, Dubai, United Arab Emirates; ^2^ Wellcome-MRC Cambridge Stem Cell Institute, Jeffrey Cheah Biomedical Center, Cambridge Biomedical Campus, Cambridge, United Kingdom; ^3^ Department of Oncology, University of Cambridge, Cambridge, United Kingdom; ^4^ Center for Genomic Discovery, Mohammed Bin Rashid University of Medicine and Health Sciences, Dubai, United Arab Emirates; ^5^ Al Jalila Genomics Center, Al Jalila Children’s Hospital, Dubai, United Arab Emirates

**Keywords:** neuroblastoma, super-enhancers, retinoic acid, differentiation, treatment-resistance

## Abstract

Neuroblastoma is a pediatric tumour that accounts for more than 15% of cancer-related deaths in children. High-risk tumours are often difficult to treat, and patients’ survival chances are less than 50%. Retinoic acid treatment is part of the maintenance therapy given to neuroblastoma patients; however, not all tumours differentiate in response to retinoic acid. Within neuroblastoma tumors, two phenotypically distinct cell types have been identified based on their super-enhancer landscape and transcriptional core regulatory circuitries: adrenergic (ADRN) and mesenchymal (MES). We hypothesized that the distinct super-enhancers in these different tumour cells mediate differential response to retinoic acid. To this end, three different neuroblastoma cell lines, ADRN (*MYCN* amplified and non-amplified) and MES cells, were treated with retinoic acid, and changes in the super-enhancer landscape upon treatment and after subsequent removal of retinoic acid was studied. Using ChIP-seq for the active histone mark H3K27ac, paired with RNA-seq, we compared the super-enhancer landscape in cells that undergo neuronal differentiation in response to retinoic acid versus those that fail to differentiate and identified unique super-enhancers associated with neuronal differentiation. Among the ADRN cells that respond to treatment, *MYCN*-amplified cells remain differentiated upon removal of retinoic acid, whereas *MYCN* non-amplified cells revert to an undifferentiated state, allowing for the identification of super-enhancers responsible for maintaining differentiation. This study identifies key super-enhancers that are crucial for retinoic acid-mediated differentiation.

## Introduction

Neuroblastoma is the most common extracranial pediatric tumour accounting for more than 15% of cancer-related deaths in children (Cheung and Dyer, 2013; Louis and Shohet, 2015). Clinical manifestations associated with this malignancy are broad and range from spontaneously regressing localized tumours to highly unfavorable metastatic tumours that relapse. Even after intensive multi-modal therapy, the overall survival of patients with high-risk disease is less than 50% (Smith and Foster, 2018). Therapeutic failure is attributed to the development of drug resistance and relapse to more aggressive tumours (Southgate et al., 2020).

A crucial aspect of neuroblastoma pathogenesis is the failure of cells to differentiate during normal sympathoadrenal development (Tomolonis et al., 2018). Retinoic acid, a vitamin A derivative and an essential factor during normal embryonic development, is used as part of the maintenance therapy to drive residual neuroblastoma cells to differentiate (Maden, 2007; Smith and Foster, 2018; Bayeva et al., 2021). However, tumours frequently relapse after developing resistance to retinoic acid (Matthay et al., 1999; Chlapek et al., 2018). Even though retinoic acid treatment has been used for decades, the exact molecular mechanism of retinoic acid-mediated differentiation is not fully understood, making it more challenging to define the mechanism of resistance to treatment.

Multiple studies have demonstrated the role of super-enhancers in controlling and defining cell identity and regulating lineage differentiation during normal development (Hnisz et al., 2013; Whyte et al., 2013). Super-enhancers are large clusters of transcriptional enhancers which define cell identity by driving the expression of critical genes. During cancer development, cancer cells can acquire super-enhancers near key oncogenes, such as MYC and TAL1 (Hnisz et al., 2013; Herranz et al., 2014; Mansour et al., 2014), and several oncogenic super-enhancers have been found across a broad spectrum of cancers (Sengupta and George, 2017; He et al., 2019). Therefore, targeting super-enhancers presents a promising therapeutic approach for cell-specific repression of oncogenes and can potentially complement other therapies (Lovén et al., 2013; Nilson et al., 2015). For instance, it has been shown that *MYCN*-overexpressing neuroblastoma cells were selectively susceptible to CDK7 inhibition by suppressing their MYCN-associated super-enhancers-associated genes (Chipumuro et al., 2014).

Core regulatory circuitries (CRCs) are formed by a small group of transcription factors that regulate the complex and dynamic mechanisms of cell lineage and identity by forming a feed-forward autoregulatory loop regulating their own expression as well as that of other target genes (Boyer et al., 2005; Saint-André et al., 2016). Super-enhancers drive the expression of these CRC factors. In neuroblastoma, two phenotypically distinct subtypes of neuroblastoma cells—adrenergic (ADRN) and mesenchymal (MES), have been identified based on the super-enhancer landscape and associated core transcriptional regulatory circuitries (CRC) (Boeva et al., 2017; van Groningen et al., 2017). It was recently demonstrated that in *MYCN*-amplified neuroblastoma cell lines, retinoic acid induces reprogramming of the ADRN CRC, leading to massive downregulation of *MYCN* expression, tumour suppression, and cell differentiation (Zimmerman et al., 2021).

In this study, we aimed to identify super-enhancers crucial for differentiation in response to retinoic acid, as well as super-enhancers that are drivers of resistance. Three different neuroblastoma cell lines of ADRN *MYCN*-amplified, ADRN *MYCN* non-amplified, and MES subtype cells were treated with all-trans-retinoic acid (ATRA) and the super-enhancer landscape after treatment and upon subsequent removal of retinoic acid was studied. We also looked at the accompanying transcriptional changes to correlate the changing super-enhancer landscape with the transcriptional response to retinoic acid. As a result, we have identified key super-enhancers associated with retinoic acid-mediated neuronal differentiation. Furthermore, by identifying core regulatory transcription factors associated with ATRA-induced differentiation, we have uncovered potential targets that may further our understanding of tumorigenesis and also in comprehending how some tumours remain resistant to differentiation therapy.

## Methods

### Cell lines and culture conditions

Neuroblastoma cell lines, SK-N-BE (2) C, SH-SY5Y, and SH-EP cells (kindly gifted by Prof. Deborah Tweddle, Newcastle University) were grown in DMEM/F-12 with GlutaMAX^™^ supplement, 10% Fetal Bovine Serum (Gibco), and 100 units/ml penicillin and 100 μg/ml.

### All-trans-retinoic acid treatment

Cells were treated with 10 µM ATRA, and the serum concentration was reduced gradually to a final concentration of 1%. The duration of treatment varied for the different cell lines, which was determined by when a homogenous neuronal cell population was observed in responsive cell lines. SK-N-BE (2) C and SH-SY5Y were treated for 10 and 14 days, respectively, whereas the resistant SH-EP cells were treated for 7 days even though no neuronal cells were observed. In all cases, the media was refreshed every 48 h. For SH-SY5Y, we followed the protocol developed by Shipley et al. (2016) with slight modifications. After initial treatment with 10 µM ATRA and reducing serum concentration on day 6, neuronal cells were trypsinized and plated to ATRA-containing neurobasal medium supported with ECM, B27, BDNF, and cAMP. For reversion, after obtaining a homogenous differentiated population in the responsive cell lines, media was refreshed to remove ATRA, and cells were grown in DMEM/F12 media with serum (10%) for 96 h.

### Immunofluorescence

Cells were treated with either DMSO or ATRA, and then fixed with 4% paraformaldehyde. After permeabilization with TritonX-100 (0.1%), cells were incubated with primary antibody (anti-beta III Tubulin, Abcam) overnight at 4°C, washed with PBST (phosphate-buffered saline, 0.1% tween 20), and then incubated with Alexa Fluor 488 conjugated secondary antibody (Abcam) for 1 h at room temperature. DAPI (4′,6-diamidino-2-phenylindole) (Sigma Aldrich) was used for counter-staining. Fluorescent images were taken with Olympus IX53 inverted microscope.

### ChIP-seq

ChIP-seq was performed as previously described (Ali et al., 2020). Briefly, Cells were fixed with 1% formaldehyde, lysed, and the DNA was sheared to 100- to 300-bp fragments. Histone-bound DNA was precipitated using H3K27ac antibody (Abcam, Cat# ab4729). Cross-linking was reversed, and DNA was purified using the Qiagen PCR purification kit (Qiagen) and quantified with the Qubit HS assay kit (Invitrogen). ChIP-seq experiments were performed in three biological replicates. First, input and pulled-down DNA were used to generate sequencing libraries according to the manufacturer’s procedure (NEBNext^®^ Ultra^™^ II DNA Library Prep Kit; Illumina). Next, the DNA was end polished, dA tailed, and adaptors with barcodes were ligated. Finally, the fragments were amplified and quantified with KAPA quantification kit (Roche), and the library size was determined using tape station (Agilent).

Libraries were sequenced on the NovaSeq 6000 in a 150 bp paired-end run to a depth of at least 25 million mapped reads per sample. Reads were trimmed using TrimGalore 0.6.4 (https://github.com/FelixKrueger/TrimGalore) to remove sequencing adaptors and poor-quality base calls, using a minimum Phred score cut-off of 20. Trimmed reads were aligned to the hg19 genome with Bowtie2 2.4.1 (Langmead and Salzberg, 2012). Unmapped reads, improperly paired reads, reads with> 4 mismatches, or insert size >2000 bp were removed. BigWig files were generated by combining bam files from biological replicates and subsampling to 160 million reads before converting to BigWig format. H3K27ac peaks were called for each replicate-input pairing, using Macs2 2.2.7.1 (Zhang et al., 2008) in narrow peak mode, using the following options: -f BAMPE -g hs--SPMR--qvalue 0.05--keep-dup all. Super-enhancer regions were identified from Macs2 peaks using ROSE 0.1 (Lovén et al., 2013; Whyte et al., 2013) with a stitching distance of 12.5 kb and without removal of TSS regions. Super-enhancer regions present in at least 2 biological replicates (with a minimum 1 bp overlap), for each treatment group within each cell line were retained as high-confidence super-enhancers. A final list of consensus super-enhancers was generated using DiffBind 2.14.0 (Ross-Innes et al., 2012; Stark and Brown, 2012) containing all possible super-enhancer regions from each condition and cell line. Super-enhancers were linked to predicted target genes within 50 kb, based on a significant positive correlation between H3K27ac at the super-enhancer and expression of the potential target gene. If no genes within 50 kb were significantly correlated, the closest gene was assigned as the regulatory target. Quantification of H3K27ac signal at super-enhancers was normalized in DiffBind by library size. ChromHMM 1.23 (Ernst and Kellis, 2012) was used to test 200 bp genomic bins for the presence or absence of H3K27ac ChIP-seq signal, and a multivariate Hidden Markov Model approach with eight states was used to predict the patterns of H3K27ac change between DMSO treated (control), ATRA treated, and ATRA withdrawn conditions for each cell line. Super-enhancers were grouped by their predominant H3K27ac state in each cell line, based on the density of windows belonging to each state (super-enhancer length/number of 200 bp windows of that state). Super-enhancers assigned to ChromHMM state groups were further grouped by their overall pattern of change between control and ATRA treated conditions into those that gained or lost H3K27ac signal after treatment and excluding those that were unchanged. CRC networks were predicted for each cell line and treatment condition using CRCmapper (Saint-André et al., 2016) with an expression cut-off of 33 and extension length 500.

### RNA-seq

Total RNA was extracted using RNeasy Mini kit (Qiagen Inc.), according to the manufacturer’s procedure. RNA was quantified using Qubit RNA kit (Invitrogen), and the integrity of RNA was determined in Tape station (Agilent). Strand-specific mRNA sequencing libraries were prepared using Illumina Stranded mRNA Library Prep Kit (Illumina), following the manufacturer’s procedure. The fragments were amplified and quantified with KAPA quantification kit (Roche), and the library size was determined using tape station (Agilent). The experiments were performed in three biological replicates for each cell line. RNA libraries were sequenced on the NovaSeq 6000 in a 150 bp paired-end run to a depth of approximately 20 million reads per library. Reads were trimmed using TrimGalore 0.6.4 (https://github.com/FelixKrueger/TrimGalore) to remove sequencing adaptors and poor-quality base calls, using a minimum Phred score cut-off of 20. Trimmed reads were aligned to the hg19 genome with STAR_2.6.1d (Anders and Huber, 2010) and quantified using the quantMode option. Differentially expressed genes were identified using DEseq2 1.30.0 (Love et al., 2014) padj < 0.05, with the following contrasts performed for each cell line: ATRA treated vs. DMSO treated (control), ATRA withdrawn vs. ATRA treated, ATRA withdrawn vs. DMSO treated.

### Bioinformatics

Principal component analysis (PCA) and sample-to-sample distance heatmaps were plotted using DiffBind normalized count data using default DiffBind settings for ChIP-seq and VST normalized data in DESeq2 RNA-seq data. Gene set enrichment analysis (GSEA) was performed with fgsea 1.160 in R using Reactome Pathway Database gene sets, or Gene Ontology (GO) Biological Process (BP) gene sets. Pathways with an adjusted *p*-value < 0.05 were considered significantly enriched. Gene set overrepresentation analysis was performed using ClusterProfiler 3.18.1 in R. GO BP or Reactome gene sets reaching adjusted *p*-value (Benjamini-Hochberg adjusted) < 0.05 were considered significant. Heatmaps showing normalized gene expression or H3K27ac signal were plotted with pheatmap 1.0.12 in R, unless stated otherwise values were row z-scaled within each cell line to allow comparison of treatment effect rather than absolute levels of expression or H3K27ac. Kaplan-meier event-free survival curves grouped by gene expression were generated on the R2: Genomics Analysis and Visualization Platform (http://r2.amc.nl), using the RPM normalized SEQC dataset (*n* = 498, GSE62564) in scan cut-off mode. CRC transcription factor binding motif frequency matrices were downloaded from the JASPAR database (Castro-Mondragon et al., 2021) and plotted using motifStack 1.34.0 in R. CRC TF motif occurrences in super-enhancers were identified using CRCmapper as described above, and circos plots generated with circlize 0.4.14 in R.

## Results

### The super-enhancer landscape is altered upon retinoic acid treatment

**FIGURE 1:**
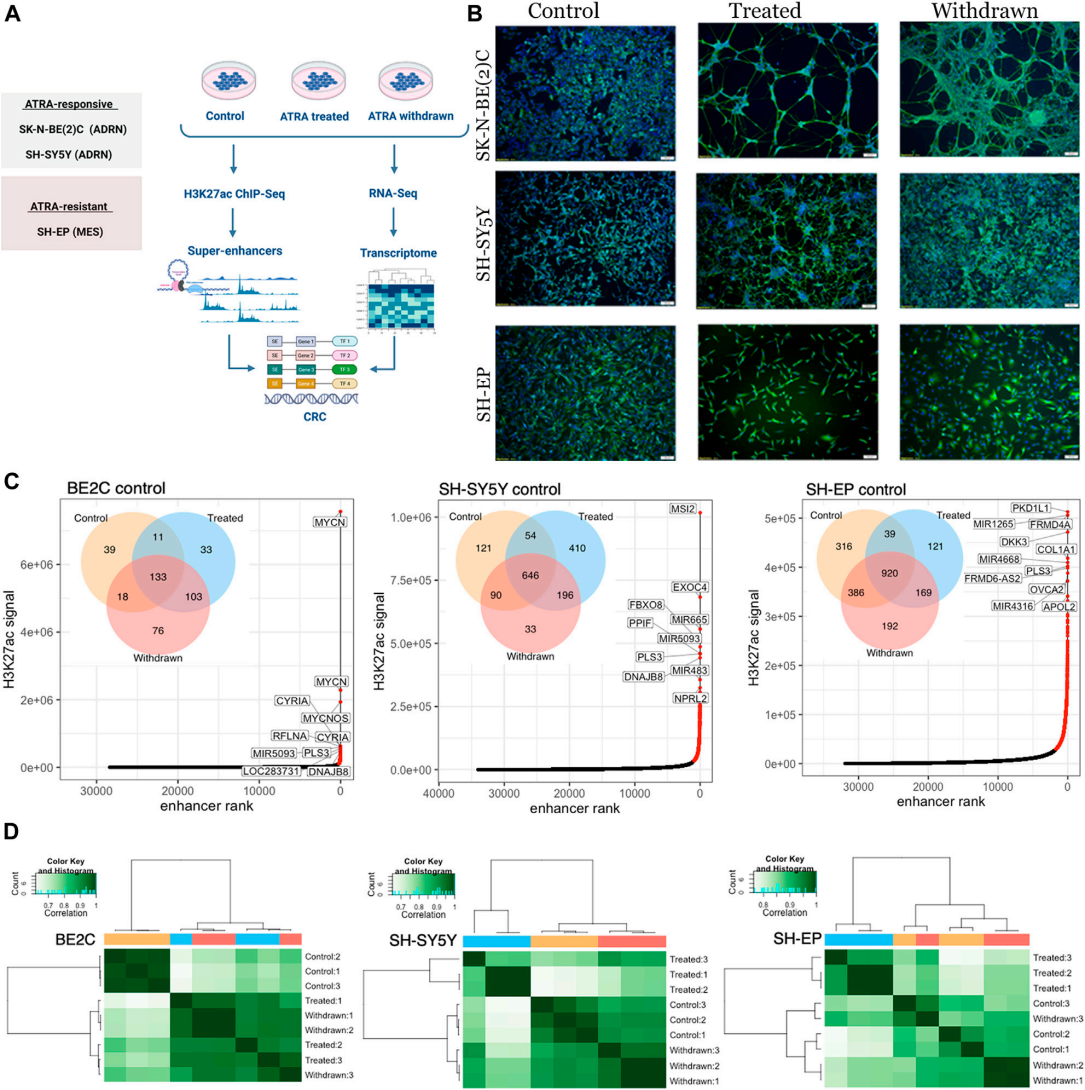
Super-enhancer landscape correlates with the observed morphological changes. **(A)** Schematic depiction of the study design. Created with BioRender.com. **(B)** Immunofluorescence staining of SK-N-BE (2) C, SH-SY5Y and SH-EP using the neuronal marker β-tubulin (*TUBB3*) showing differentiation in ATRA responsive cell lines [SH-SY5Y and SK-N-BE (2) C]. In the ATRA resistant cell line (SH-EP), neuronal projections were absent. Cell nuclei were counterstained with DAPI (blue). Scale bar indicates 100 µm **(C)** Super-enhancers identified in control conditions (highlighted in red) were ranked according toH3K27ac signal, for BE2C, SH-SY5Y, and SH-EP. The top 10 ranked super-enhancers are annotated with their predicted regulatory target. Venn diagrams show the number of super-enhancers identified in control, ATRA treated and withdrawn samples for the corresponding cell lines. **(D)** Correlation matrix showing hierarchical clustering of H3K27ac ChIP-seq samples. Heat map displaying Pearson correlations between pairwise comparisons for all conditions, in each cell line. Pearson correlations were calculated using the normalized read depth across consensus super-enhancers identified in any condition, for each cell line.

To investigate the role of super-enhancers in neuroblastoma differentiation and to identify differentiation-associated super-enhancers in response to ATRA treatment, we subjected three phenotypically and genetically different neuroblastoma cell lines: *MYCN*-amplified ADRN cell line, SK-N-BE (2) C (BE2C), *MYCN* non-amplified ADRN cell line SH-SY5Y, and *MYCN* non-amplified MES cell line SH-EP, to treatment with ATRA. We carried out morphological analysis and ChIP-seq for the activating histone modification H3K27ac after treatment of cells with ATRA (“treated”) and upon subsequent withdrawal of ATRA (“withdrawn”), compared to the DMSO (“control”) (Figure 1A).

To induce differentiation, neuroblastoma cells were treated with retinoic acid in low serum media (Kovalevich and Langford, 2013). Initially, we assessed the differentiation response of each cell line to ATRA treatment and withdrawal by immunofluorescence staining using the neuronal marker β-tubulin (*TUBB3*). A differentiated neuronal population with axon-like processes expressing *TUBB3* was observed in BE2C and SH-SY5Y cells (Figure 1B, Supplementary Figure S1A), indicating sensitivity to ATRA-induced differentiation as previously reported (Shipley et al., 2016). However, SH-EP cells did not develop neuronal processes in response to ATRA treatment, in line with their reported resistance to ATRA treatment (Edsjö et al., 2004) (Figure 1B, Supplementary Figure S1A). After obtaining a differentiated population, or in the case of SH-EP cells, after 7-days of treatment, ATRA was removed, and cells were grown in media with serum for 96 h. Interestingly, BE2C cells retained a differentiated morphology upon withdrawal of treatment, whereas in SH-SY5Y, the neurite projections were no longer present (Figure 1B, Supplementary Figure S1A, Supplementary Movies S1, S2). During ATRA treatment, cell proliferation was restricted, and proliferation resumed upon ATRA withdrawal (Supplementary Figure S1B).

Next, we performed ChIP-seq for H3K27ac and used the ROSE algorithm to identify super-enhancers in each cell line across the three different conditions (Hnisz et al., 2013; Lovén et al., 2013; Whyte et al., 2013). ATRA treatment and subsequent withdrawal altered the super-enhancer landscape in all three cell lines studied (Figure 1C). Although the most highly ranked super-enhancers (based on total H3K27ac signal) generally remained unchanged across the treatment conditions (Supplementary Figure S1C), *de novo* establishment and loss of super-enhancers were observed upon treatment in all three cell lines, indicating ATRA-induced changes in the super-enhancer landscape (Figure 1C, Supplementary Figure S1C). Interestingly, the most highly ranked super-enhancer in BE2C under control conditions, MYCN, showed a loss of H3K27ac signal upon treatment with ATRA, which remained low upon withdrawal of ATRA. The MYCN locus is highly amplified in BE2C cells, and MYCN amplification is associated with more aggressive disease in patients (Harenza et al., 2017; Otte et al., 2021). Notably, despite their resistance to ATRA-induced differentiation, we observed significant changes in the super-enhancer landscape in SH-EP cells as a result of ATRA treatment. We noticed that ATRA withdrawal did not fully restore the super-enhancer profile to that of the control conditions in any cell lines, suggesting that ATRA treatment has more prolonged effects on the super-enhancer landscape of neuroblastoma cells that persist beyond treatment withdrawal (Figure 1C, Supplementary Figure S1C). Hierarchical clustering and principal component analysis (PCA) of super-enhancer regions showed that SH-EP and SH-SY5Y revert towards their untreated baseline state upon ATRA removal, whereas in the BE2C cells the super-enhancer landscape of ATRA treated and withdrawn cells are highly similar (Figure 1D, Supplementary Figure S1D). This finding is consistent with the phenotypic changes observed in cell morphology (Figure 1B, Supplementary Figure S1A). These ATRA-induced changes remain more stable in BE2C cells compared to SH-SY5Y, 96 h after ATRA withdrawal.

### Differential transcriptional changes in response to retinoic acid treatment and withdrawal are reflective of the phenotypic changes

**FIGURE 2:**
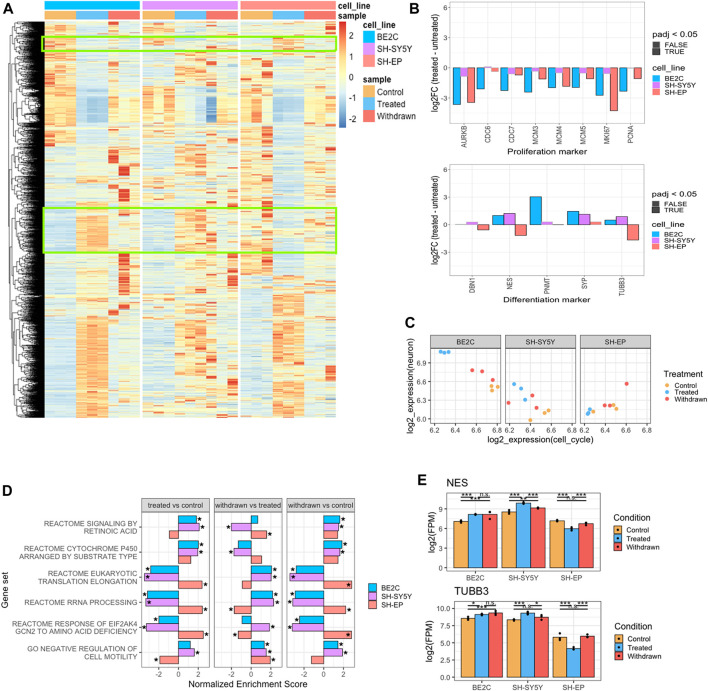
Transcriptomic changes corroborate the morphological observations. **(A)** Heatmap showing the expression pattern of genes differentially expressed between control and ATRA treated conditions, identified in any cell line (padj <0.05, LogFC >2, *n* = 3). Green boxes indicate genes that show opposite patterns of expression between responsive and resistant cell lines. Values are z-scaled by row within each cell line. **(B)** Barplots showing the expression (log2 fold change) of proliferation and neuronal differentiation markers between control and ATRA treated conditions. Significantly differentially expressed genes (padj < 0.05, *n* = 3) bordered in black, non-significant bordered in grey. **(C)** Dotplots showing the average expression (log2 FPM) of cell cycle and neuron associated gene modules for each cell line and treatment condition (*n* = 3). **(D)** Gene set enrichment analysis (GSEA) showing selected significantly enriched Reactome Pathway and GO BP gene sets when comparing expression between control and ATRA treated samples (treated vs. control), ATRA treated and withdrawn samples (withdrawn vs. treated), and between control and withdrawn samples (withdrawn vs. control). Bars show the normalised enrichment score for each cell line, positive scores indicate gene set upregulation, negative scores indicate downregulation. Significantly enriched gene sets (padj < 0.05) marked by asterisk **(E)** Bar plots showing the expression of *NES* and *TUBB3* in control, treated and withdrawn conditions in responsive cell lines (BE2C and SH-SY5Y) and resistant cell line (SH-EP). Statistical significance determined using DESeq2 (*padj < 0.05, **padj < 0.01, and ***padj < 0.001).

To identify the transcriptomic changes associated with ATRA treatment, we performed RNA-seq under the conditions described in Figure 1A (DMSO control, ATRA treated, and following ATRA withdrawal). PCA of the RNA-seq data indicated that ATRA treatment and subsequent withdrawal has a consistent genome-wide effect on the transcriptome (Supplementary Figure S2A). Since the cell lines used in our study belong to ADRN/MES class of neuroblastoma cell lines, we checked the expression of selected ADRN and MES markers which were reported previously (Boeva et al., 2017; van Groningen et al., 2017). As expected, ADRN markers were highly expressed in BE2C and SH-SY5Y whereas MES markers were highly expressed in SH-EP (Supplementary Figure S2B). We did not see any consistent changes in the expression pattern of markers across ATRA treatment and withdrawal except for *ASCL1*, *HAND2*, and *IRF1*. Additionally, we looked at the expression of retinoic acid markers, *RARA* and *RARB*, and saw that their expression upregulated upon treatment in ATRA-responsive cell lines (BE2C and SH-SY5Y) but not in SH-EP (Supplementary Figure S2C). Incidentally, expression of these markers downregulated in SH-SY5Y but not in BE2C upon reversion further confirming that the retinoic acid response in BE2C is more stable compared to SH-SY5Y. Whilst some ATRA-responsive genes followed a common pattern of change upon treatment and subsequent withdrawal, we also identified cell line-specific responsive and resistant genes (Figure 2A, Supplementary Figure S2D,E). In line with the reduced proliferation on ATRA treatment (Supplementary Figure S1B), we observed downregulation of proliferation markers upon treatment with ATRA (Figure 2B). In addition, neuronal differentiation markers such as *PNMT*, *SYP*, *NES*, and *TUBB3* were upregulated in the responsive cell lines, BE2C and SH-SY5Y. In contrast, differentiation markers were downregulated in the resistant cell line, SH-EP (Figure 2B).

To better assess the degree to which ATRA treatment affects differentiation at the transcriptional level, we defined two gene modules to measure neuronal identity and cell cycle activity by calculating the average expression of genes annotated with the GO Cellular Component terms neuron projection (GO:0043005)/neuron cell body (GO:0043025), or mitotic spindle (GO:0072686)/mitotic checkpoint complex (GO:0033597), respectively. We found that ATRA treatment led to upregulation of the neuronal module and downregulation of the cell cycle module in responsive cell lines to varying degrees. This appears to be most robust in BE2C and weaker in SH-SY5Y (Figure 2C). In contrast, there was no activation of the neuronal module in SH-EP, indicating its inability to undergo neuronal differentiation in response to ATRA treatment. On subsequent withdrawal of ATRA, proliferation resumed in all cell lines at the transcriptional level (Figure 2C, Supplementary Figure S1B), mirroring what was observed phenotypically.

To gain more insight into the functional role of the significantly enriched or repressed genes upon ATRA treatment (treated vs. control) and subsequent withdrawal (withdrawn vs. treated), we performed gene set enrichment analysis (GSEA). We also directly compared control and withdrawn conditions (withdrawn vs. control). We found that upregulated genes were significantly enriched for association with retinoic acid signaling in ATRA responsive cell lines (BE2C and SH-SY5Y), as well as cytochrome p450 enzymes which regulate ATRA metabolism, and interleukin 10 signaling—a known target of the retinoic acid signaling pathway (Figure 2D treated vs. control). In contrast, repressed genes were associated with protein synthesis in responsive cell lines, whereas these terms were positively enriched in SH-EP. Retinoic acid signaling pathway genes were significantly positively enriched in SH-SY5Y and BE2C cell lines, however, in SH-EP cell, this pathway was not enriched (Figure 2D treated vs. control), suggesting that ATRA resistant cells fail to activate the retinoic acid signaling pathway in response to ATRA treatment. Interestingly, the same pathways that were upregulated in SH-EP upon treatment appear to be downregulated in SH-SY5Y (Figure 2D). SH-EP (S-type cells) and SH-SY5Y (N-type cells) are derived from the same parental SK-N-SH cell line; however, the different cell identity reinforced by the super-enhancer landscape is mirrored in the different ways by which each cell type responds to ATRA, which further suggests that regulation by super-enhancers has a role to play in the differential response of neuroblastoma cell lines to retinoic acid. It is important to note that the retinoic acid signaling pathway is positively enriched in SH-EP upon withdrawal of ATRA, compared to the ATRA treated condition (Figure 2D withdrawn vs. treated). In contrast, this pathway is down-regulated in the responsive SH-SY5Y cell line upon withdrawal but remains activated in BE2C (Figure 2D withdrawn vs. treated). This suggest that activation of retinoic acid signaling is not stably maintained in both the responsive cell lines, although even in SH-SY5Y retinoic acid signaling pathway genes remain positively enriched compared to control conditions (Figure 2D withdrawn vs. control) after 96 h of withdrawal, suggesting some level of maintenance or a slow decrease in retinoic acid signaling. This further indicates that BE2C could also revert to the control state after a longer duration, consistent with what was shown recently (Zimmerman et al., 2021). Notably, the normalized enrichment score measuring retinoic acid signaling pathway gene set downregulation is greater in SH-SY5Y than BE2C, reflecting the observation that SH-SY5Y cells retract ATRA-induced neuronal projections and revert, whereas BE2C maintain their differentiated morphology.

Furthermore, we found that neuronal differentiation markers TUBB3 and NESTIN (NES) were up-regulated in response to ATRA treatment in responsive cell lines, and this activation is stably maintained after ATRA withdrawal in BE2C cells (Figure 2E). Finally, since it has recently been shown that retinoic acid signaling is required for motility (van Groningen et al., 2021), we looked at the cell motility gene set. Interestingly, the class “negative regulation of cell motility” is repressed in SH-EP cells suggesting cell motility is promoted by ATRA treatment and suppressed again on withdrawal, whereas this is not the case in BE2C and SH-SY5Y cells (Figure 2D) (van Groningen et al., 2021).

### Expression of target genes correlated with gained or lost super-enhancers

**FIGURE 3:**
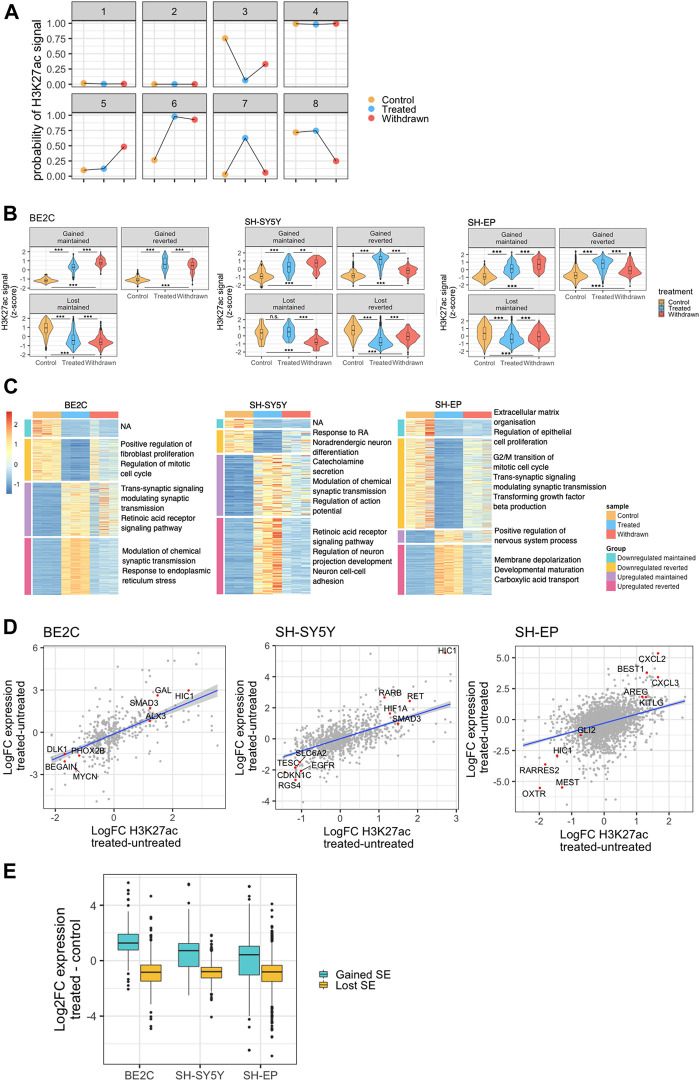
Expression of target genes correlates with gained or lost super-enhancers. **(A)** Representation of the eight states modeled by ChromHMM for identifying patterns in super-enhancer changes across the three conditions. Y-axis shows the ChromHMM emissions results, which represents the probability of observing H3K27ac in that treatment condition, conditioned by the state to which it has been assigned. **(B)** Violin plots showing changes in the normalised H3K27ac signal (row z-score) at ATRA responsive super-enhancers identified for each NB cell line, and grouped by patterns of H3K27ac change. Statistical significance determined by One-Way ANOVA followed by Tukey’s post hoc test (*p < 0.05, **p < 0.01, and ***p < 0.001). **(C)** Heatmaps showing row z-score normalised expression of retinoic acid treatment response genes (DESeq2 treated vs. untreated, padj <0.05, log2FoldChange >2). Genes were split into those significantly up or downregulated in treated samples compared to untreated. These two groups were subsequently split into those significantly down or upregulated, respectively, between treated and withdrawn samples, and those that showed no significant change between treated and withdrawn samples. Each gene group is annotated with relevant significantly enriched GO BP terms (padj <0.05). **(D)** Dot plots showing the correlation between log2FC H3K27ac at ATRA responsive super-enhancers and the log2FC expression of their predicted regulatory targets. Selected positively correlated targets are highlighted (red) and annotated with their target gene. **(E)** Box plots showing the log2FC in expression of genes associated with a super-enhancer that is gained or lost as a result of ATRA treatment.

To identify the pattern of changes in the super-enhancer landscape in response to ATRA treatment and withdrawal, we used ChromHMM. This tool employs a multivariate Hidden Markov Model to model the presence or absence of a chromatin mark in genomic bins (200 bp). We used ChromHMM to predict an 8-state model of H3K27ac state changes between our three conditions and used this information to designate each super-enhancer as gained, lost, or unchanged (Figure 3A) (more detailed information in methods). We found consensus super-enhancers for each cell line, i.e., super-enhancers identified in at least 2 of the 3 replicates for each treatment and combined to include super-enhancers present in any of the three treatment conditions. To designate each super-enhancer to a state, we found the density of 200 bp sub-regions of each state in a super-enhancer and assigned the state with the highest density as the predominant state for that super-enhancer. We then calculated the normalized H3K27ac signal in each consensus super-enhancer region for each state and grouped them based on their pattern of H3K27ac over the three treatment conditions.

We developed this method because super-enhancers defined by the ROSE algorithm can consist of long stretches of the genome, with regions of high H3K27ac signal punctuated by large valleys with low signal. This makes it challenging to apply differential analysis methods developed for RNA-seq, such as DEseq2, which can be applied to other types of count data assuming a negative binomial distribution, including ChIP-seq datasets (Love et al., 2014). The large valleys dilute the H3K27ac signal, and the presence of multiple peaks in a single super-enhancer that could change in opposite directions confounds the ability to identify changing super-enhancers.

To investigate the super-enhancers specifically involved in the ATRA response, we looked at four main patterns of ATRA responsive super-enhancers based on those predicted by the model: gained maintained, gained reverted, lost maintained, and lost reverted. The “maintained” group includes super-enhancers that were either “gained” or “lost” upon ATRA treatment but with no significant change upon withdrawal. The “reverted” group, refers to super-enhancers that were either “gained” or “lost” upon ATRA treatment, but were significantly changed after withdrawal of ATRA (Figure 3B, Supplementary Figure S3). We found that these four patterns were represented in the SH-SY5Y cell line; however, in BE2C, and SH-EP cell lines, there were no super-enhancers that were lost upon ATRA treatment and regained after withdrawal, perhaps reflecting the relative stability of the BE2C differentiated phenotype even after withdrawal of treatment (Figure 3B). We also grouped the transcriptome similarly into four distinct groups. The heat maps in Figure 3C reveal how the transcriptome changes across the different conditions in each cell line. GO analysis of the gene sets of each group showed results consistent with phenotypic observations. Notably, the retinoic acid receptor signaling pathway upregulated upon treatment in both SH-SY5Y and BE2C and is maintained in BE2C upon ATRA withdrawal, whereas in SH-SY5Y, cells that phenotypically revert, this pathway gets downregulated. Interestingly, in SH-EP cells, we see an enrichment of the GO process “regulation of nervous system process” suggesting some minor activation of neuronal pathways, however, “response to retinoic acid” is downregulated, suggesting that the downregulation of retinoic acid signaling has a significant role in the resistance of SH-EP cells to ATRA (Figure 3C).

Super-enhancers can modulate the expression of their target genes. Therefore, we used a correlation-proximity-based method to identify the regulatory target genes of the identified super-enhancers (Whyte et al., 2013). As detailed in the methods section, active genes within 50 kb of the super-enhancer with a significant positive correlation between super-enhancer strength and gene expression level were assigned as putative targets. As a result, we found a significant correlation between the changes in H3K27ac at super-enhancers and the expression of putative target genes such as the transcription factor *HIC1* (Figure 3D). Interestingly, in responsive cell lines, ATRA treatment led to a gain of H3K27ac signal at the *HIC1* super-enhancer as well as increased *HIC1* expression. In contrast, H3K27ac decreased at the *HIC1* super-enhancer region in the resistant SH-EP cells, resulting in *HIC1* repression. HIC1 has been described as a tumour suppressor and is frequently hyper-methylated in cancers, including neuroblastoma (Rathi et al., 2003; Zhang et al., 2010). In response to ATRA treatment, the gain of H3K27ac positively correlates with the activation of putative regulatory targets, and loss of super-enhancer acetylation is associated with target gene downregulation (Figure 3E). These changes suggest that super-enhancers gained/lost upon ATRA treatment cause significant changes in the expression of their associated target genes.

### Identification of gained or lost super-enhancers in responsive cell lines

**FIGURE 4:**
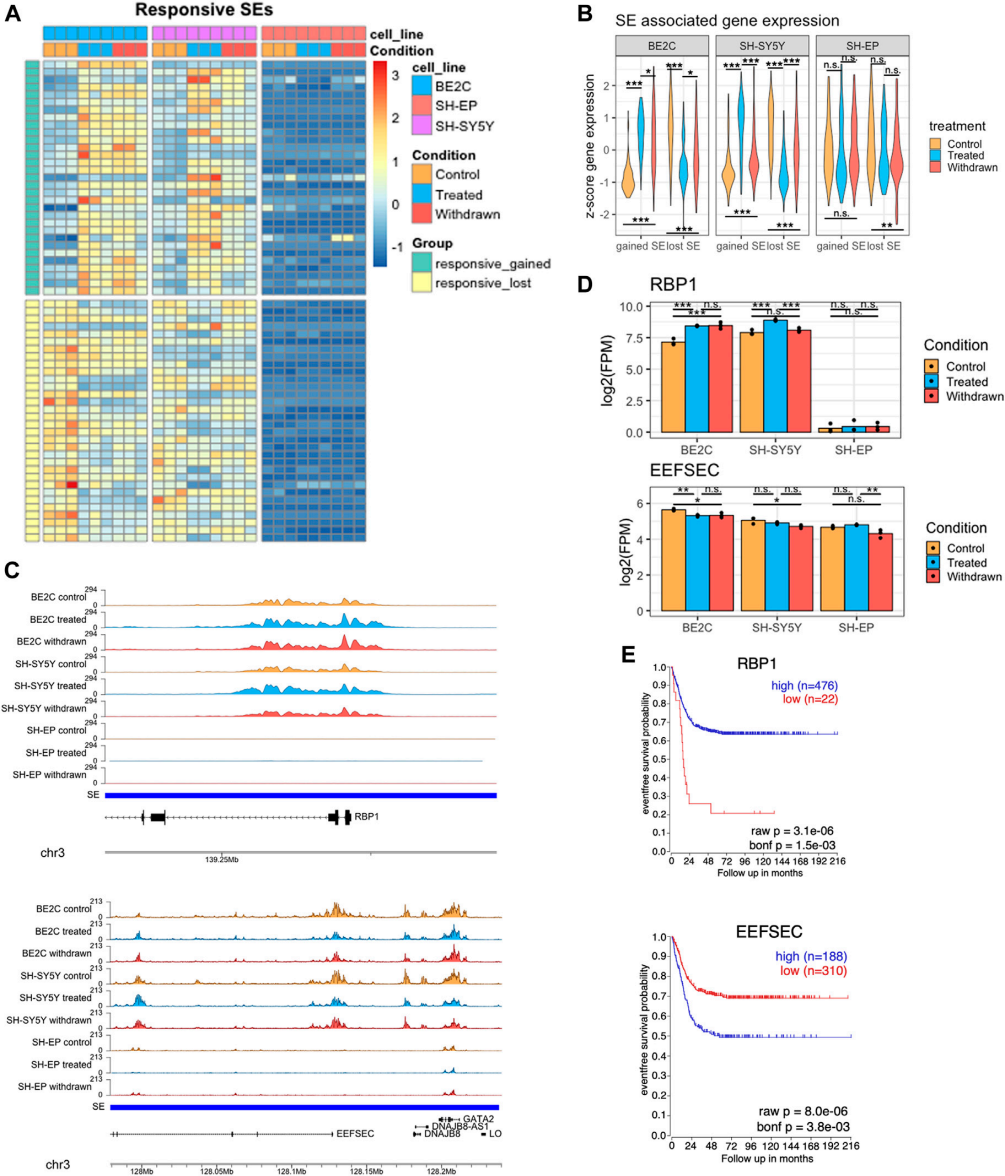
Identification of super-enhancers differentially expressed in responsive cell lines **(A)** Heatmap showing the changes in normalised H3K27ac signal at super-enhancers which are gained or lost in responsive cell lines after treatment with ATRA, but remain unchanged in SH-EP. Row z-score calculated across all cell lines. **(B)** Violin plots showing the z-score expression of genes associated with the set of gained/lost SEs shown in panel **(A)**, in responsive and resistant cell lines. Statistical significance determined by One-Way ANOVA followed by Tukey’s post hoc test (*p < 0.05, **p < 0.01, and ***p < 0.001). **(C)** Representative ChIP-seq tracks displaying gained (upper panel) and lost (lower panel) super-enhancer regions identified in ATRA responsive cell lines. **(D)** Bar plots showing the expression of *RBP1* and *EEFSEC* in control, treated, and withdrawn conditions in responsive cell lines (BE2C and SH-SY5Y) and resistant cell line (SH-EP). Statistical significance determined using DESeq2 (*padj < 0.05, **padj < 0.01, and ***padj < 0.001). **(E)** Kaplan-Meier survival curves showing the probability of event free survival in two groups of neuroblastoma patients, split based on their tumour expression level of *RBP1* or *EEFSEC*.

To identify super-enhancers associated with ATRA-induced differentiation in responsive cell lines, we compared the super-enhancer profile of the responsive and resistant cell lines upon treatment. We specifically looked at the super-enhancers gained or lost in the cell lines that differentiated in response to ATRA (BE2C and SH-SY5Y) but remained absent or unchanged in SH-EP (Figure 4A, Supplementary Figure S4A). The failure to gain or lose H3K27ac at these super-enhancers in SH-EP may be responsible for the lack of differentiation in response to ATRA. Most of these gained and lost super-enhancers are absent in SH-EP, pointing to the inactivity of pathways required for the differentiation response to ATRA. We found significant changes in the expression of putative target genes associated with gained and lost super-enhancers upon treatment and removal of ATRA, suggesting these are functional super-enhancers (Figure 4B). Once again, our data suggest that the BE2C cell line maintains the ATRA-induced changes to super-enhancer associated gene expression even after ATRA withdrawal, whereas SH-SY5Y reverts towards the control state (Figure 4B).

Next, we looked more closely at specific candidate ATRA responsive super-enhancers with documented relevance to retinoic acid signaling or neuronal differentiation. We identified several gained super-enhancers associated with genes involved in retinol uptake, and neuronal differentiation related to early development, such as *RBP1* and *KAT6B*, suggesting that ATRA treatment may induce neuroblastoma differentiation by promoting the activation of key super-enhancer networks associated with cell differentiation and development. Retinol binding protein 1 (RBP1) is a cytoplasmic retinol-binding protein and tumour suppressor involved in retinol uptake and storage (Napoli, 2012). A super-enhancer spans the RBP1 gene with the H3K27ac signal concentrated in a 14 kb region over RBP1 exons 1 and 2 (Figure 4C upper panel). ATRA treatment leads to further H3K27ac deposition at this region, correlated with significant upregulation of RBP1 mRNA in differentiating ATRA responsive cell lines (Figure 4D). In contrast, this super-enhancer is absent in the resistant SH-EP line, and RBP1 is expressed at very low levels, with no significant change upon ATRA treatment (Figure 4C,D upper panel).

KAT6B encodes a histone acetyltransferase and a MOZ/MORF protein complex component. A super-enhancer associated with KAT6B was gained upon treatment in responsive cell lines with a corresponding upregulation in its expression. In contrast, in SH-EP there was no super-enhancer identified at this region, and KAT6B gene expression was not affected by ATRA treatment. (Supplementary Figures S4B,C). To explore whether these super-enhancer-associated genes activated by ATRA treatment in responsive lines had prognostic significance, we conducted Kaplan-Meier analysis using the R2: Genomics Analysis and Visualization Platform (http://r2.amc.nl). High tumour expression of *RBP1*, or *KAT6B* is associated with improved patient survival outcomes (Figure 4E, Supplementary Figure S4D).

H3K27ac enrichment over genes associated with lost super-enhancers, such as *EEFSEC*, was significantly reduced in responsive cell lines upon ATRA treatment (Figure 4C lower panel). This was reflected in the downregulation of their expression levels in responsive cell lines (Figure 4D). EEFSEC is a selenocysteine tRNA-specific eukaryotic elongation factor that has a critical role during embryonic development (Zhang et al., 2017; Liu et al., 2021). Importantly, lower expression of *EEFSEC* in patient tumours correlates with improved survival outcomes suggesting the significance of this gene in neuroblastoma tumour biology (Figure 4E). The *EEFSEC* super-enhancer was not present in SH-EP cells.

### Super-enhancers that maintain the ATRA treated state after withdrawal of treatment are associated with stable ATRA-induced differentiation

**FIGURE 5:**
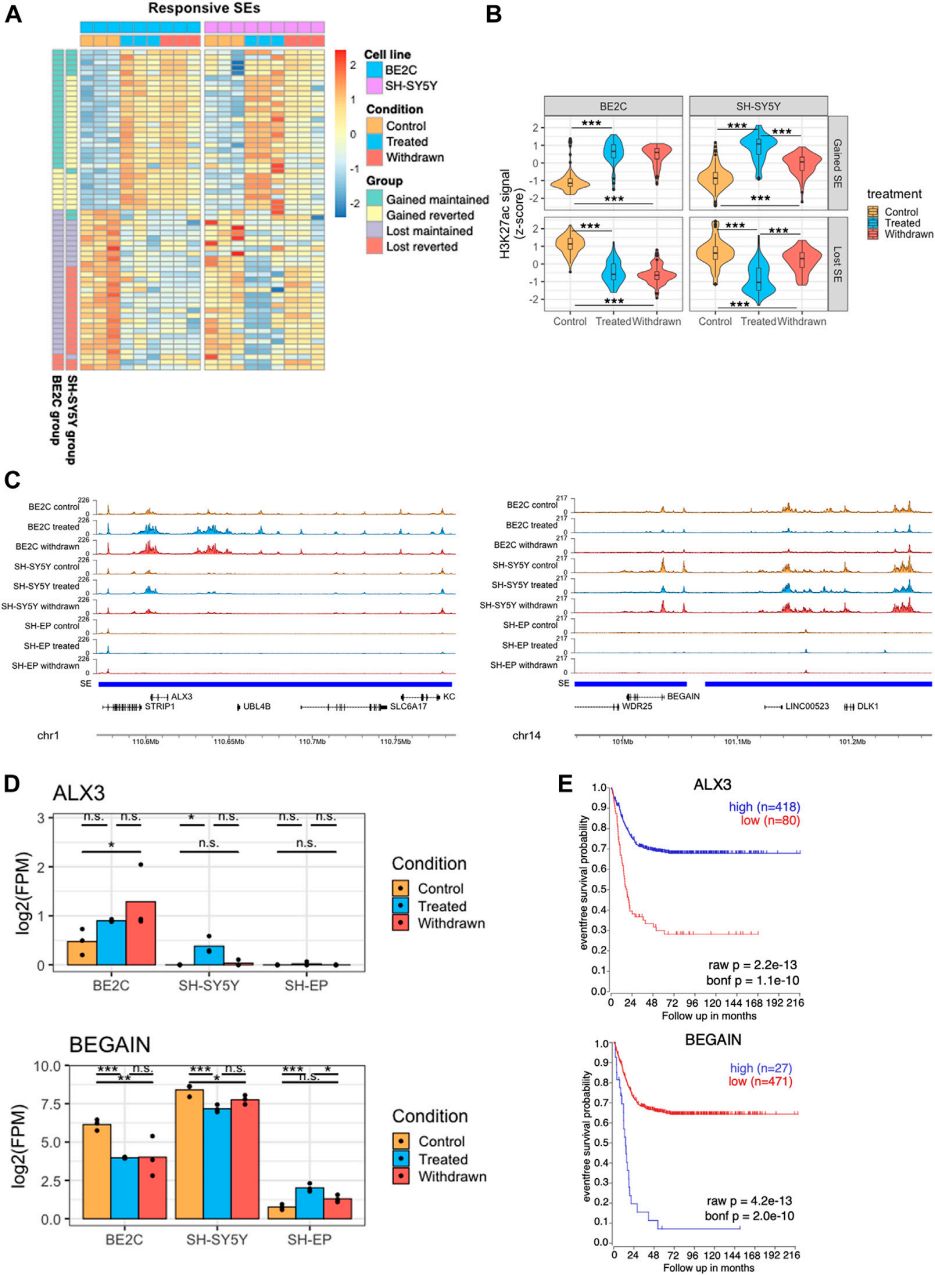
Super-enhancers that are stably maintained in BE2C are associated with differentiation. **(A)** Heatmap showing changes in H3K27ac levels at super-enhancers which are responsive to ATRA treatment in both BE2C and SH-SY5Y cell lines. Data is z-scaled for each super-enhancer region, within each cell line. Data was split into four groups for each cell line, based on the pattern of H3K27ac after ATRA treatment and withdrawal. **(B)** Violin plots summarizing the H3K27ac signal data shown in panel **(A)**. Statistical significance was calculated by one-way ANOVA followed by Tukey’s post-hoc testing (***p < 0.001, **p < 0.01, unlabeled = not significant). **(C)** Representative ChIP seq tracks showing H3K27ac signal at super-enhancer regions which gain (left panel) or lose (right panel) H3K27ac on treatment with ATRA, and are subsequently maintained in BE2C but not SH-SY5Y cells. **(D)** Bar plots showing the expression of *ALX3* and *BEGAIN* in control, treated and withdrawn conditions in responsive cell lines (BE2C and SH-SY5Y) and resistant cell line (SH-EP). Statistical significance determined using DESeq2 (*padj < 0.05, **padj < 0.01, and ***padj < 0.001). **(E)** Kaplan-Meier survival shows the significance of high/low expression of *ALX3*, and *BEGAIN* in event-free survival in neuroblastoma.

Our observations imply that ATRA treatment induced differentiation of SH-SY5Y and BE2C cells plausibly by the gain and loss of super-enhancers, and by regulating the expression of associated target genes. However, these responsive cell lines behave differently after withdrawal of ATRA; BE2C cells start proliferating but retain their differentiated phenotype, whereas SH-SY5Y cells return to their original undifferentiated state (Figure 1B, Supplementary Movies S1, S2). This led us to investigate to what extent ATRA-induced changes to the super-enhancer landscape are maintained after its withdrawal and if this correlates with differences in behaviour between the two responsive cell lines. In the SH-SY5Y cell line, H3K27ac deposited at gained super-enhancers during ATRA treatment was reduced after ATRA withdrawal, as compared to the stably differentiated BE2C line, where H3K27ac levels at gained super-enhancers were more likely to be maintained or reduced only slightly, indicating a lesser degree of reversion to the pre-treatment state (Figures 5A,B; Supplementary Figures S5A,B). In BE2C cells, 23 out of the 31 gained super-enhancers maintained ATRA-induced levels of H3K27ac after withdrawal, compared to only 8/33 in SH-SY5Y (Supplementary Figure S5C). This effect was even more apparent when looking at super-enhancers lost after ATRA treatment. In SH-SY5Y, super-enhancers tended to regain H3K27ac and revert to pre-treatment levels; however, in BE2C, there was no significant change after ATRA withdrawal (Figures 5A,B, Supplementary Figure S5D). In BE2C cells, only 3/31 reverted the ATRA-induced changes compared to SH-SY5Y, where 19 out of the 29 lost super-enhancers reverted (Supplementary Figure S5C).

Predicting that the super-enhancers which were maintained in BE2C upon withdrawal are associated with neuronal differentiation, we analyzed the expression of selected super-enhancer-associated genes. *ALX3*, a neuronal differentiation-associated gene, was predicted to be regulated by one such super-enhancer (Figure 5C left panel). ALX3 is a homeobox transcription factor implicated in differentiation and development and is reported to be hypermethylated in advanced-stage neuroblastomas (Wimmer et al., 2002). *ALX3* expression was upregulated on ATRA treatment in both differentiating cell lines but not in SH-EP cells. Upon withdrawal, *ALX3* is further upregulated in BE2C but not in SH-SY5Y, suggesting its significance in ATRA-induced neuronal differentiation (Figure 5D). Moreover, high expression of *ALX3* is associated with better survival in neuroblastoma patients further implicating the significance of this gene as a critical regulator in neuroblastoma differentiation (Figure 5E).

Another super-enhancer associated gene *BEGAIN*, identified in our study, is predicted to be involved in regulating postsynaptic neurotransmitter receptor activity (Deguchi et al., 1998; Llères et al., 2019). Low tumour expression is associated with better survival in neuroblastoma patients, and incidentally, upon ATRA treatment, we found *BEGAIN* expression is downregulated in differentiating cell lines but not in SH-EP, where it is upregulated (Figure 5C–E right panel). Furthermore, upon withdrawal, the expression reverts in SH-SY5Y but not in BE2C cells. This data suggests that activation of specific super-enhancers leads to differentiation in neuroblastoma cells.

### HIC1 and SMAD3 are part of the ATRA responsive CRC of neuroblastoma cell lines

Our analyses identified several transcription factors as super-enhancer-associated targets, which appear to be activated by ATRA treatment in responsive cell lines, including HIC1, SMAD3, RARB, and HIF1A (Figure 3D). We also saw the suppression of established ADRN core transcription regulatory circuitry (CRC) components PHOX2B, and MYCN in BE2C cells, which stabilize the ADRN CRC in MYCN-amplified cell lines (Supplementary Figure S6A) (Durbin et al., 2018; Zeid et al., 2018; Zimmerman et al., 2021). This prompted us to investigate the effect of ATRA treatment on the CRC of responsive neuroblastoma cell lines. We aimed to identify a CRC associated with the ability to differentiate in response to ATRA and determine whether a new CRC is established in ATRA differentiated cells.

**FIGURE 6:**
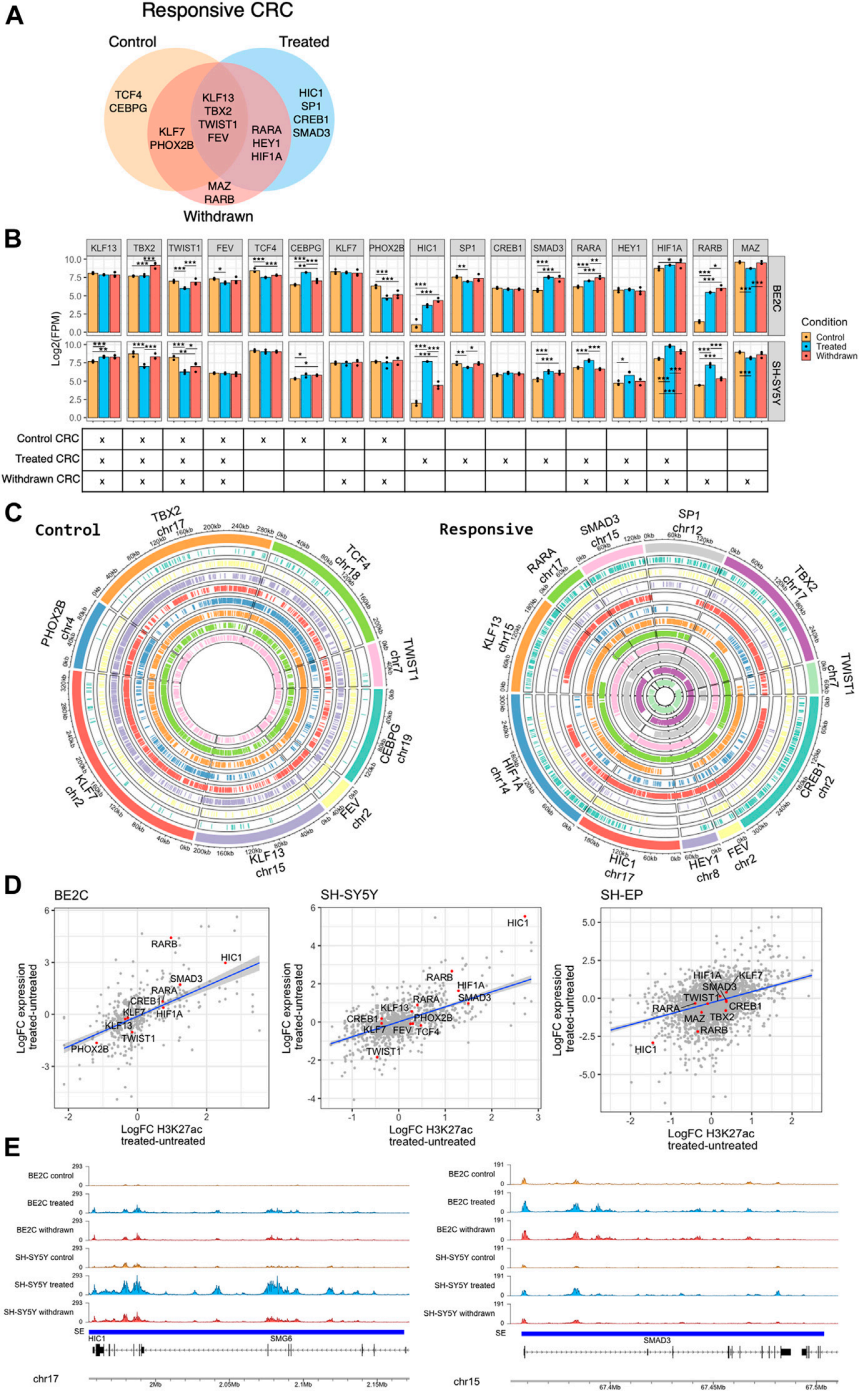
HIC1 and SMAD3 are part of the responsive CRC of neuroblastoma cell lines. **(A)** Venn diagram showing the transcription factors predicted in the core transcription regulatory circuitry (CRC) of responsive cell lines (BE2C and SH-SY5Y) under control, ATRA treated, and withdrawn conditions. **(B)** Bar plots showing the expression levels of predicted responsive CRC transcription factors under control, ATRA treated, and withdrawn conditions in BE2C and SH-SY5Y. Statistical significance determined using DESeq2 (*padj < 0.05, **padj < 0.01, and ***padj < 0.001) (top panel). The CRC(s) to which each transcription factor belongs is marked below its bar plot (bottom panel). **(C)** Circos plots showing the presence of binding motifs of the CRC components, identified as part of control (left panel) and ATRA treated responsive (right panel) CRC, in their corresponding super-enhancer regions. Each color corresponds to the labelled CRC transcription factor and the binding motifs of the CRC factor are marked in the corresponding super-enhancer region. **(D)** Dot plots showing the positive correlation between expression of CRC components and H3K27ac signal at super-enhancers which are gained or lost in responsive cell lines (BE2C and SH-SY5Y). **(E)** ChIP-seq track view of H3K27ac signal at HIC1 (left panel) and SMAD3 (right panel) super-enhancers in BE2C and SH-SY5Y.

Employing CRCmapper (Saint-André et al., 2016), we first predicted the CRC of each cell line under each condition (Supplementary Figure S6B). Then, to identify CRC components that define ATRA responsiveness, we identified the regulators in both BE2C and SH-SY5Y cells (Figure 6A). The resulting ATRA responsive CRC contained previously reported ADRN CRC components, including PHOX2B, TBX2, and KLF7 (Boeva et al., 2017; Durbin et al., 2018; Zimmerman et al., 2021), as well as novel candidates CEBPG, KLF13, TWIST1, and FEV, which were identified in the responsive cell lines but not in the ATRA-resistant SH-EP cell line (Figure 6A, Supplementary Figure S6B). According to Boeva et al. (2017) and van Groningen et al. (2017), KLF7 is a member of the ADRN CRC. However, our data suggest that KLF7 is a CRC component in the MES identity SHEP line (Supplementary Figure S6B). The identified CRCs may prime the epigenome and transcriptome landscape to enable ATRA-mediated differentiation of neuroblastoma cells.

Next, we looked at the effect of ATRA treatment on the identified ATRA responsive CRC. The expression level of the individual regulators of the CRC was analyzed from the RNA-seq data. In all treatment conditions, a core set of transcription factors (KLF13, TBX2, TWIST1, and FEV) were maintained as CRC components, with relatively stable super-enhancers and expression levels (Figure 6B). However, ATRA treatment established a new CRC, no longer including TCF4 and CEBPG or the ADRN TFs PHOX2B, but now driven by core TFs including Retinoic Acid Receptor Alpha (RARA), HIC1, amongst others (Figure 6A). Consistent with the above data, we show that the binding motifs of the individual CRC transcription factor components (in control and treated states) were found in the identified super-enhancer regions (Figure 6C, Supplementary Figure S6C). Expression of the CRC components correlated mainly with the associated gain/loss of H3K27ac levels at super-enhancers; however, as expected, this was only the case in responsive cell lines (Figure 6D). In contrast, ATRA-induced CRC factors were largely unaffected by ATRA treatment in SH-EP (resistant cell line).

We observed *HIC1*, *SMAD3*, *RARA*, and *RARB* were significantly upregulated upon ATRA treatment and became part of the ATRA-induced CRC either on treatment with ATRA or after its subsequent withdrawal (Figures 6A,B)*.* HIC1 (Hypermethylated In Cancer 1) is a tumour suppressor, and transcriptional repressor reported to be silenced in medulloblastoma by hypermethylation (Briggs et al., 2008). *HIC1* and SMAD3 are upregulated in responsive cell lines upon treatment (Figure 6B). Moreover, upon withdrawal of ATRA, their activation is maintained in BE2C cells, which retain a differentiated phenotype, but returns to control levels in SH-SY5Y cells which revert to a neuroblastic morphology on ATRA withdrawal, suggesting its significance as a specific differentiation-associated transcription factor (Figures 6B,E). HIC1 was identified as a master regulator/cofactor of human neuronal fate specification in a CRISPR activation screen and was also shown to have an essential role in embryogenesis (Black et al., 2020; Ray and Chang, 2020). Similar to HIC1, SMAD3 also play a crucial role in embryogenesis. SMAD3 has a vital role in the TGF-β signaling pathway, and SMAD3 activity reduces the expression of progenitor proteins and promotes activation of neuronal differentiation by supporting cell cycle exit (García-Campmany and Martí, 2007). SMAD3 has also been shown to activate neural differentiation in concert with histone demethylase JMJD3 in chick spinal cord (Estarás et al., 2012).

Our findings suggest that ATRA-induced differentiation of responsive neuroblastoma cell lines is mediated by the deposition and maintenance of H3K27ac at existing and *de novo* super-enhancer regions at key transcription factor genes such as *HIC1* and *SMAD3* to bring about neuronal differentiation.

### ATRA resistant SHEP cells suppress RAR expression and fail to modulate differentiation-associated super-enhancers in response to treatment

**FIGURE 7:**
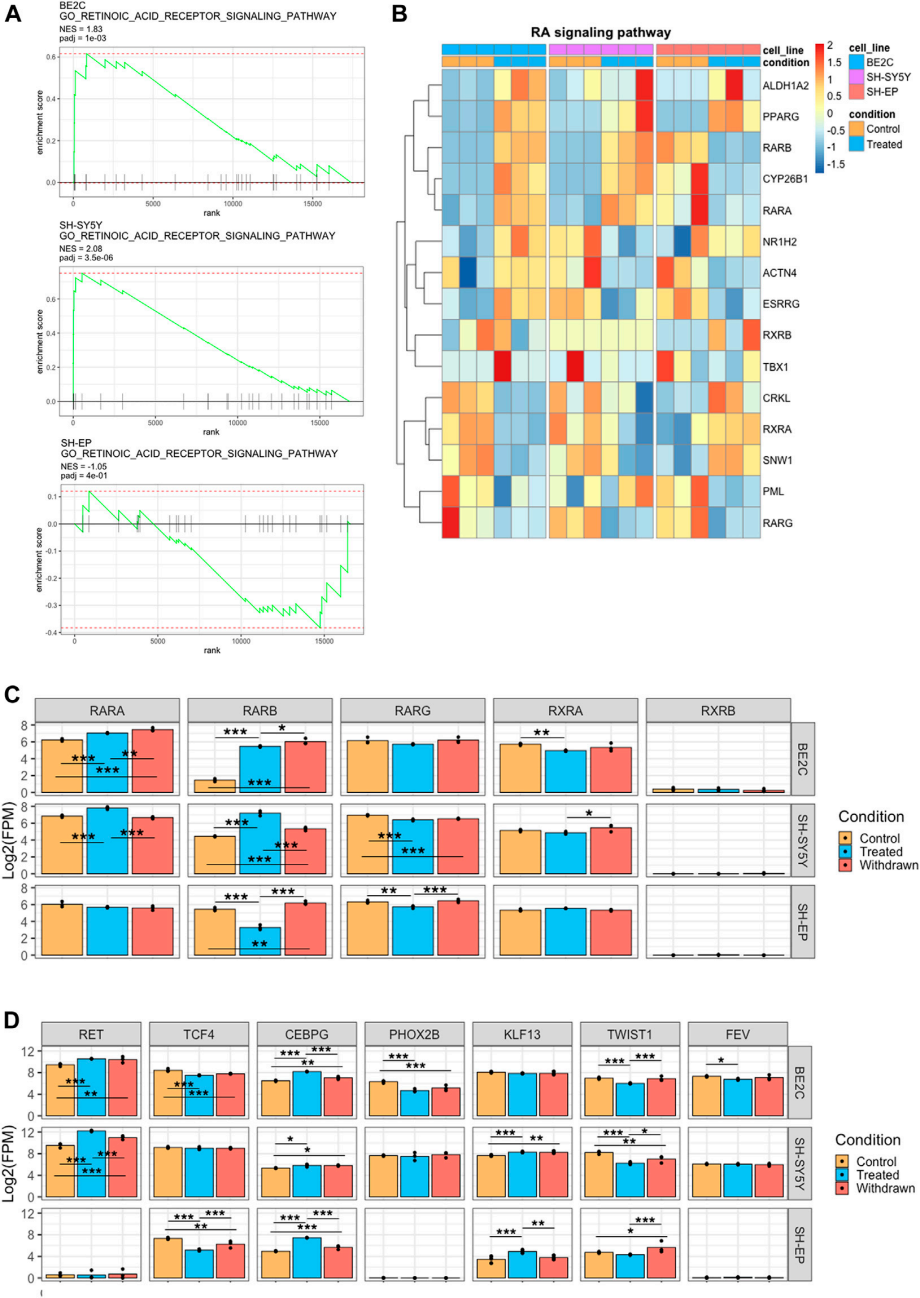
ATRA resistance in SH-EP is due to suppressed retinoic acid signaling and failure to activate the differentiation associated super-enhancers. **(A)** GSEA enrichment plots showing the normalised enrichment score (NES) of the retinoic acid receptor signaling pathway gene set, comparing control and ATRA treated conditions for each cell line. **(B)** Heatmaps showing the change in expression of retinoic acid signaling pathway genes between control and treated conditions, row z-scaled within each cell line. **(C)** Bar plots showing the normalised expression of retinoic acid receptor gene homologs. Statistical significance determined using DESeq2 (*padj < 0.05, **padj < 0.01, and ***padj < 0.001) **(D)** Bar plots showing the normalised expression of CRC transcription factor genes. Statistical significance determined using DESeq2 (*padj < 0.05, **padj < 0.01, and ***padj < 0.001).

In addition to identifying the differentiation-associated super-enhancers and their target regulators, we also wanted to investigate how SH-EP cells fail to differentiate upon ATRA treatment. We hypothesized that the super-enhancer landscape plays a role in regulating ATRA resistance in MES cells. Our transcriptomic data indicates that the retinoic signaling pathway is repressed in SH-EP cells upon ATRA treatment; however, the genes associated with this pathway are significantly activated in BE2C and SH-SY5Y (Figure 2D). We found no significant transcriptional change in retinol metabolism or retinoic acid response in SH-EP cells by GSEA. In contrast, retinoic acid metabolism and response genes were highly enriched in responsive cell lines after treatment with ATRA (Figure 7A). Genes in the retinoic signaling pathway were differentially regulated in responsive and resistant cell lines upon treatment (Figure 7B). Hierarchical clustering of the retinoic acid response genes showed that SH-EP and SH-SY5Y cluster together under control conditions. However, SH-SY5Y and BE2C cluster together upon ATRA treatment, indicating a common response in these lines (Supplementary Figures S7A,B).

All three cell lines expressed the retinoic acid receptors *RARA*, *RARG*, and *RXRA* to similar levels under control conditions, indicating they all possess the capacity to affect transcriptional regulation through RAR activity (Figures 7B,C). However, on treatment with ATRA, both *RARA* and *RARB* expression were significantly upregulated in both responsive cell lines, whereas this is not the case in the resistant SH-EP cells in which *RARB* was significantly repressed (Figures 7B,C). Importantly, we found that super-enhancer associated with *RARB* expression is gained in responsive cell lines, whereas a slight decrease was observed in the resistant SH-EP (Supplementary Figure S7C). Additionally, we found that SH-EP cells do not express *RET,* a tyrosine kinase receptor, which was previously reported as critical for retinoic acid-induced differentiation (Oppenheimer et al., 2007). In contrast, in responsive lines, *RET* is highly expressed and marked by a super-enhancer (Figure 7D, Supplementary Figure S7C). Similarly, super-enhancers that are specifically associated with differentiation such as ALX3 and JARID2 were specifically observed in responsive cell lines but not in SH-EP (Figure 5C, Supplementary Figure S7C). JARID2 (Jumonji- and ARID-domain-containing protein) is essential in the differentiation of stem cells and embryonic development (Pasini et al., 2010).

Since CRC transcription factors are critical in regulating transcriptional responses, we compared the CRC components which drive the transcriptional landscape in ATRA responsive and resistant cells under control and treatment conditions. We found some similarities in transcriptional response between gene expression associated between resistant and responsive CRCs, with both containing TCF4, KLF7, and TBX2 as members (Supplementary Figure S6B). TCF4 and TCF2 are selective dependency genes for neuroblastoma according to CRISPR KO screens (depmap.org), indicating they are necessary for survival and may explain their conservation between various neuroblastoma lines (Figure 7D). However, the SH-EP control CRC lacked the transcription factors CEBPG, PHOX2B, KLF13, TWIST1, and FEV found in ATRA responsive lines under control conditions (Figure 6A, 7D, Supplementary Figure S6B).

Intriguingly, our data shows that expression of retinoic acid signaling components are regulated in opposite directions by ATRA treatment, e.g., RARB is activated by treatment in responsive lines but repressed in SH-EP (Figures 7B,C). This, alongside a recent study showing that MES cells endogenously produce retinoic acid to promote cell motility (van Groningen et al., 2021), suggests that the retinoic acid signaling pathway is constitutively active in MES type cells. We found that even though retinoic acid signaling pathway components are expressed in SH-EP cells, the differentiation-associated super-enhancers such as ALX3 are absent. The absence of these differentiation-associated super-enhancers possibly renders the cells resistant to differentiation even in the presence of an active retinoic acid signaling pathway.

## Discussion

Neuroblastoma is purported to arise due to a differentiation block during the development of normal sympathoadrenal system (Matthay et al., 2016). Sympathoadrenal progenitors destined to form chromaffin and sympathetic neuronal cells become arrested in an immature state, leading to sustained proliferative capacity, differentiation arrest, and malignancy. Therefore, understanding the process of differentiation and what causes differentiation arrest is vital in developing better therapeutic approaches.

Super-enhancers and the aberrant expression of associated oncogenes have been reported in many pediatric cancers, including neuroblastoma (Lovén et al., 2013; Chipumuro et al., 2014). Recent studies have shown how super-enhancers and the associated CRC components maintain a proliferative state and control neuroblastoma tumours (Boeva et al., 2017; van Groningen et al., 2017). In this study, we used a retinoic acid-mediated differentiation system to identify the key super-enhancers and the associated core transcription factors that can induce differentiation in neuroblastoma cells. Retinoic acid plays an important role in early embryo development and neurodevelopment (Bayeva et al., 2021). It regulates the cell cycle, and loss of retinoic acid signaling is associated with de-differentiation and cancer development (Janesick et al., 2015). Furthermore, it is well established that high levels of retinoic acid are essential to induce cell proliferation arrest and drive differentiation of human neuroblastoma cell lines (Sidell et al., 1983; Reynolds et al., 2003); therefore, impaired retinoic acid signaling has been implicated in human neuroblastoma.

In our study, we used three cell lines, two that are reported to be susceptible to retinoic acid-mediated differentiation, the responsive (BE2C and SH-SY5Y) and the non-differentiating resistant (SH-EP) cell line (Edsjö et al., 2004). Using this approach, we were able to identify super-enhancers involved explicitly in mediating retinoic acid differentiation. Consistent with the previous studies, differentiation was observed only in responsive lines (BE2C and SH-SY5Y) when treated with retinoic acid; however, all three cell lines (BE2C, SH-SY5Y, and SH-EP) showed reduced proliferation. In contrast to a recent study that suggested retinoic acid is required for MES-type cells to proliferate (van Groningen et al., 2021), our data shows downregulation of proliferation in both ADRN and MES cell lines upon treatment, which may also be attributed to the low serum used in our treatment conditions.

It is important to note that the *MYCN*-amplified BE2C cells retained the differentiated state even after the removal of retinoic acid; however, the other two cell lines reverted to the control phenotype, which is corroborated by the changes in their super-enhancer landscapes. Interestingly, this provided more stringent selection criteria for super-enhancers associated with differentiation than ATRA-responsiveness alone. The reversion of the differentiated phenotype upon withdrawal of ATRA is significant in the context of relapse tumors. Stable maintenance of gained super-enhancers requires master transcription factors, chromatin remodellers, and histone modifiers (Wang et al., 2019). Therefore, a combination treatment, including HDAC or DNA methylation inhibitors and retinoic acid drugs, would be a promising strategy in treating high-risk neuroblastoma (Rettig et al., 2015; Westerlund et al., 2017).

The expression of genes associated with neuronal differentiation reflects the morphological changes, suggesting the importance of super-enhancers in retinoic acid-mediated differentiation. Upon ATRA withdrawal, the expression of genes associated with “retinoic acid signaling” pathway reverts in SH-SY5Y but not in BE2C cells (Figure 3C), consistent with the more stable retention of differentiated phenotype of BE2C compared to SH-SY5Y. This is probably attributed to the active retinoic acid signaling pathway in BE2C cells that affects the downstream targets that help retain the differentiated state. Additionally, our data indicated that SH-SY5Y and SH-EP cells, although derived from the same parental cell line, behaved differently upon ATRA treatment, further validating our hypothesis that the presence or absence of super-enhancers is significant in controlling differentiation. Our approach helped us identify super-enhancers and associated genes that could plausibly be important in neuronal differentiation and the ATRA response. Interestingly, many of the identified genes associated with differentiation-specific super-enhancers, such as ALX3 and HIC1 have a hypermethylated promoter that causes repression of their expression (Lai et al., 1999; Kuppumbatti et al., 2000; Jerónimo et al., 2004; Toki et al., 2010). We speculate that ATRA treatment leads to the activation of super-enhancers that modulate the downstream targets leading to their active transcription.

Our data shows that retinoic acid has different effects on super-enhancers in responsive and resistant cell lines. The impact on super-enhancers appears to be functional as there are correlated changes in the expression of the CRC transcription factors. A recent study identified that retinoic acid treatment disrupts the adrenergic CRC in *MYCN*-amplified cell line and reprograms it into a “retino-sympathetic” CRC, resulting in rapid down-regulation of *MYCN* expression coupled with the induction of cell differentiation and proliferative arrest (Zimmerman et al., 2021). Here, by using responsive cell lines consisting of both *MYCN*-amplified and non-amplified, we were able to identify consensus CRC components associated with retinoic acid-mediated differentiation. Upon treatment with ATRA, the responsive CRC shifts away from reliance on ADRN neuroblastoma markers, and a new CRC associated with the ATRA response is assembled (Figure 6A). Notably, the CRC members identified include the retinoic acid receptor homologs RARA, RARB and the tumour suppressors HIC1 and SMAD3 (Millet and Zhang, 2007; Janeckova et al., 2015). HIC1 and SMAD3 are modulators of Wnt, TGF-β signaling pathways, which are essential in neuronal differentiation (Meyers and Kessler, 2017; Becker and Wilting, 2018; Ray and Chang, 2020). Functional studies of these regulators can help elucidate their role in the differentiation of sympathoadrenal precursors during embryonic development.

Resistance to retinoic acid and relapse is a major problem when using the drug as maintenance therapy (Masetti et al., 2012; Chlapek et al., 2018). Previous studies have identified different mechanisms of resistance, such as hypermethylation of CRABPII promoter, c-MYC overexpression, suppression of ALDH activity by negative feedback mechanism and modulation of cytosolic retinoic acid associated enzymes that regulate concentration of retinols (Fu et al., 2012; Januchowski et al., 2013; Chlapek et al., 2018). Genes such as *ZNF423* and *NF1* have previously been identified to confer retinoic acid resistance (Huang et al., 2009; Hölzel et al., 2010). Resistance to ATRA in *MYCN*-amplified cell lines has been reported in multiple studies; however, few studies address how *MYCN* non-amplified cell lines are resistant to retinoic acid. By using the SH-EP cell line in our experiment, which are MES_type *MYCN* non-amplified cells (Boeva et al., 2017), we were able to discern the resistant mechanisms adopted by this subtype of cells. MES cells are chemoresistant and are enriched in relapsed tumours (Boeva et al., 2017; Westerhout et al., 2022).

The physiological functions of retinoic acid are exerted through the nuclear receptors, RAR-α/β/γ, and the retinoid X receptor RXR, which form a heterodimer and bind to DNA elements known as retinoic acid response elements (RARE) (Bastien and Rochette-Egly, 2004). The bound complex further associates with nuclear receptor coactivators and corepressors to regulate target gene expression. Known corepressors include NCoR, SMRT, and histone deacetylases, resulting in gene repression *via* removal of H3K27ac (Chen and Evans, 1995; Hörlein et al., 1995; Urvalek and Gudas, 2014). In addition, many RAR coactivators, such as CBP/p300, act as histone acetyltransferases to facilitate transactivation (Chen et al., 1997).

Differences in expression of RAR genes in the different cell lines upon treatment may mean that ATRA effects are mediated through different RAR/RXR heterodimers, potentially leading to differences in retinoic acid response (Figure 7B). Our study showed that the different homologs of RAR receptors are expressed differently in the responsive and resistant cell lines, suggesting that the different epigenomic and transcriptomic effects of ATRA treatment could be partly due to abnormal RAR activity. Further, our data shows that in SH-EP cells, although the genes for synapse organization and neuronal differentiation are enriched (Figure 3C), those involved in retinoic acid signaling are downregulated (Figures 3C, 7A). This signaling pathway, which includes RARA and RARB, is important for ATRA-mediated differentiation (Carpentier et al., 1997). *RARB* silencing is associated with resistance to retinoic acid in epithelial carcinogenesis and in breast and prostate tumours (Wan et al., 1999; Ren et al., 2005). Surprisingly, in SH-EP cells, *RARB* is actively expressed under control conditions and after withdrawal but is repressed upon ATRA treatment (Figure 7C). Moreover, we see that retinoic acid signaling pathway is downregulated upon ATRA treatment. It was observed that the super-enhancer associated with *RARB* had a slight decrease upon ATRA treatment, and the corresponding repression of *RARB* could result in the failure to activate downstream targets of ATRA response. The retinoic acid signaling pathway seems to be constitutively active in SH-EP cells even without ATRA treatment, however, the differentiation-specific super-enhancers identified in our study (target genes *ALX3*, *RBP1*, *JARID2*, and *RET*) are absent, rendering it unable to differentiate. Put together, the repression of retinoic signaling, mediated by a loss of *RARB* super-enhancer upon ATRA treatment, and the absence of differentiation-associated super-enhancers could contribute to SHEP’s resistance to ATRA. The important question to be asked is why the differentiation-associated super-enhancers are absent in SH-EP. It could be speculated that these cells are blocked at a different developmental stage compared to the responsive cell lines, one where the differentiation associated super-enhancers identified in this study have not yet been “primed”, and consequently are not available to respond to retinoic acid.

Importantly, ADRN/MES subtypes represent different differentiation states, with MES being undifferentiated and ADRN being more lineage-committed. Transdifferentiation between these cell identities is associated with cellular plasticity (van Groningen et al., 2017; van Groningen et al., 2019). The differentiation state of cells within a tumor and the cross-talk between the different subtypes is relevant in drug resistance and relapse (Yan et al., 2011; Lecca et al., 2018). The differential responses to retinoic acid treatment and withdrawal we observed may also reflect these divergent differentiation states. Although, we did not see consistent changes in the ADRN/MES marker expression upon ATRA treatment and withdrawal (Supplementary Figure S2B), the expression of markers across the 2 ADRN cell lines were variable in the untreated, mirroring the inherent heterogeneity between the cell lines. Both of the responsive cell lines in our study differentiated when treated with ATRA, but upon withdrawal, SH-SY5Y dedifferentiated whereas BE2C remained differentiated, suggesting that although both these cell lines are ADRN, their molecular profile emulate the different stages of sympathoadrenal lineage development and perhaps SH-SY5Y cells have a lower differentiation state compared to BE2C.

In conclusion, this study highlights the role of super-enhancers in the differential response of neuroblastoma cell lines to ATRA. We identified super-enhancers associated with neuronal differentiation under *in vitro* conditions. It is important to highlight that these *in vitro* cell culture models may not mimic the exact conditions present under *in vivo* conditions. In addition, our study used genome-wide analysis using ChIP-seq and RNA-seq for target discovery. Hence, additional studies on the functional aspects of the individual super-enhancers and associated target genes in *in vivo* models are warranted. A more thorough understanding of the regulatory role of super-enhancers and the associated targets during development of the sympathoadrenal system and in the context of disease is required before considering these studies for clinical applications.

Furthermore, in our study we identified responsive CRC factors that could be important in ATRA-mediated differentiation. Functional studies aimed at elucidating the mechanisms that suppress these responsive CRC components in neuroblastoma tumours will help devise new therapeutic approaches against this pediatric malignancy. Moreover, by delineating the resistant mechanism in SH-EP cells, we identified super-enhancers associated with RAR receptors as important in mediating resistance. Pharmacological activation of retinoic acid signaling along with mechanisms that could activate the differentiation-specific super-enhancers could possibly sensitize SH-EP cells to retinoic acid-mediated differentiation. Thus, this study helped elucidate the role of super-enhancers in retinoic acid-mediated differentiation in neuroblastoma cells.

## Data Availability

The datasets presented in this study can be found in online repositories. The names of the repository/repositories and accession number(s) can be found below: Gene Expression Omnibus accession number: GSE202241.

## References

[B1] Ali F. R. Marcos D. Chernukhin I. Woods L. M. Parkinson L. M. Wylie L. A. (2020). Dephosphorylation of the proneural transcription factor ASCL1 Re-engages a latent post-mitotic differentiation program in neuroblastoma. Mol. Cancer Res. 18 (12), 1759–1766. 10.1158/1541-7786.Mcr-20-0693 33046535 PMC7614603

[B2] Anders S. Huber W. (2010). Differential expression analysis for sequence count data. Genome Biol. 11 (10), R106. 10.1186/gb-2010-11-10-r106 20979621 PMC3218662

[B3] Bastien J. Rochette-Egly C. (2004). Nuclear retinoid receptors and the transcription of retinoid-target genes. Gene 328, 1–16. 10.1016/j.gene.2003.12.005 15019979

[B4] Bayeva N. Coll E. Piskareva O. (2021). Differentiating neuroblastoma: A systematic review of the retinoic acid, its derivatives, and synergistic interactions. J. Pers. Med. 11 (3), 211. 10.3390/jpm11030211 33809565 PMC7999600

[B5] Becker J. Wilting J. (2018). WNT signaling, the development of the sympathoadrenal-paraganglionic system and neuroblastoma. Cell. Mol. Life Sci. 75 (6), 1057–1070. 10.1007/s00018-017-2685-8 29058015 PMC5814469

[B6] Black J. B. McCutcheon S. R. Dube S. Barrera A. Klann T. S. Rice G. A. (2020). Master regulators and cofactors of human neuronal cell fate specification identified by CRISPR gene activation screens. Cell Rep. 33 (9), 108460. 10.1016/j.celrep.2020.108460 33264623 PMC7730023

[B7] Boeva V. Louis-Brennetot C. Peltier A. Durand S. Pierre-Eugène C. Raynal V. (2017). Heterogeneity of neuroblastoma cell identity defined by transcriptional circuitries. Nat. Genet. 49, 1408–1413. 10.1038/ng.3921 28740262

[B8] Boyer L. A. Lee T. I. Cole M. F. Johnstone S. E. Levine S. S. Zucker J. P. (2005). Core transcriptional regulatory circuitry in human embryonic stem cells. Cell 122 (6), 947–956. 10.1016/j.cell.2005.08.020 16153702 PMC3006442

[B9] Briggs K. J. Corcoran-Schwartz I. M. Zhang W. Harcke T. Devereux W. L. Baylin S. B. (2008). Cooperation between the Hic1 and Ptch1 tumor suppressors in medulloblastoma. Genes Dev. 22 (6), 770–785. 10.1101/gad.1640908 18347096 PMC2275430

[B10] Carpentier A. Balitrand N. Rochette-Egly C. Shroot B. Degos L. Chomienne C. (1997). Distinct sensitivity of neuroblastoma cells for retinoid receptor agonists: Evidence for functional receptor heterodimers. Oncogene 15 (15), 1805–1813. 10.1038/sj.onc.1201335 9362447

[B11] Castro-Mondragon J. A. Riudavets-Puig R. Rauluseviciute I. Berhanu Lemma R. Turchi L. Blanc-Mathieu R. (2021). JASPAR 2022: The 9th release of the open-access database of transcription factor binding profiles. Nucleic Acids Res. 50 (D1), D165–D173. 10.1093/nar/gkab1113 PMC8728201 34850907

[B12] Chen H. Lin R. J. Schiltz R. L. Chakravarti D. Nash A. Nagy L. (1997). Nuclear receptor coactivator ACTR is a novel histone acetyltransferase and forms a multimeric activation complex with P/CAF and CBP/p300. Cell 90 (3), 569–580. 10.1016/S0092-8674(00)80516-4 9267036

[B13] Chen J. D. Evans R. M. (1995). A transcriptional co-repressor that interacts with nuclear hormone receptors. Nature 377 (6548), 454–457. 10.1038/377454a0 7566127

[B14] Cheung N.-K. V. Dyer M. A. (2013). Neuroblastoma: Developmental biology, cancer genomics and immunotherapy. Nat. Rev. Cancer 13, 397–411. 10.1038/nrc3526 23702928 PMC4386662

[B15] Chipumuro E. Marco E. Christensen C. L. Kwiatkowski N. Zhang T. Hatheway C. M. (2014). CDK7 inhibition suppresses super-enhancer-linked oncogenic transcription in MYCN-driven cancer. Cell 159 (5), 1126–1139. 10.1016/j.cell.2014.10.024 25416950 PMC4243043

[B16] Chlapek P. Slavikova V. Mazanek P. Sterba J. Veselska R. (2018). Why differentiation therapy sometimes fails: Molecular mechanisms of resistance to retinoids. Int. J. Mol. Sci. 19 (1), 132. 10.3390/ijms19010132 PMC5796081 29301374

[B17] Deguchi M. Hata Y. Takeuchi M. Ide N. Hirao K. Yao I. (1998). BEGAIN (brain-enriched guanylate kinase-associated protein), a novel neuronal PSD-95/SAP90-binding protein. J. Biol. Chem. 273 (41), 26269–26272. 10.1074/jbc.273.41.26269 9756850

[B18] Durbin A. D. Zimmerman M. W. Dharia N. V. Abraham B. J. Iniguez A. B. Weichert-Leahey N. (2018). Selective gene dependencies in MYCN-amplified neuroblastoma include the core transcriptional regulatory circuitry. Nat. Genet. 50 (9), 1240–1246. 10.1038/s41588-018-0191-z 30127528 PMC6386470

[B19] Edsjö A. Nilsson H. Vandesompele J. Karlsson J. Pattyn F. Culp L. A. (2004). Neuroblastoma cells with overexpressed MYCN retain their capacity to undergo neuronal differentiation. Lab. Invest. 84 (4), 406–417. 10.1038/labinvest.3700061 14767491

[B20] Ernst J. Kellis M. (2012). ChromHMM: Automating chromatin-state discovery and characterization. Nat. Methods 9 (3), 215–216. 10.1038/nmeth.1906 22373907 PMC3577932

[B21] Estarás C. Akizu N. García A. Beltrán S. de la Cruz X. Martínez-Balbás M. A. (2012). Genome-wide analysis reveals that Smad3 and JMJD3 HDM co-activate the neural developmental program. Development 139 (15), 2681–2691. 10.1242/dev.078345 22782721

[B22] Fu Y.-S. Wang Q. Ma J.-X. Yang X.-H. Wu M.-L. Zhang K.-L. (2012). CRABP-II methylation: A critical determinant of retinoic acid resistance of medulloblastoma cells. Mol. Oncol. 6 (1), 48–61. 10.1016/j.molonc.2011.11.004 22153617 PMC5528385

[B23] García-Campmany L. Martí E. (2007). The TGFβ intracellular effector Smad3 regulates neuronal differentiation and cell fate specification in the developing spinal cord. Development 134, 65–75. 10.1242/dev.02702 17138664

[B24] Harenza J. L. Diamond M. A. Adams R. N. Song M. M. Davidson H. L. Hart L. S. (2017). Transcriptomic profiling of 39 commonly-used neuroblastoma cell lines. Sci. Data 4, 170033. 10.1038/sdata.2017.33 28350380 PMC5369315

[B25] He Y. Long W. Liu Q. (2019). Targeting super-enhancers as a therapeutic strategy for cancer treatment. Front. Pharmacol. 10, 361. 10.3389/fphar.2019.00361 31105558 PMC6499164

[B26] Herranz D. Ambesi-Impiombato A. Palomero T. Schnell S. A. Belver L. Wendorff A. A. (2014). A NOTCH1-driven MYC enhancer promotes T cell development, transformation and acute lymphoblastic leukemia. Nat. Med. 20 (10), 1130–1137. 10.1038/nm.3665 25194570 PMC4192073

[B27] Hnisz D. Abraham B. J. Lee T. I. Lau A. Saint-André V. Sigova A. A. (2013). Super-enhancers in the control of cell identity and disease. Cell 155 (4), 934–947. 10.1016/j.cell.2013.09.053 24119843 PMC3841062

[B28] Hölzel M. Huang S. Koster J. Ora I. Lakeman A. Caron H. (2010). NF1 is a tumor suppressor in neuroblastoma that determines retinoic acid response and disease outcome. Cell 142 (2), 218–229. 10.1016/j.cell.2010.06.004 20655465 PMC2913027

[B29] Hörlein A. J. Näär A. M. Heinzel T. Torchia J. Gloss B. Kurokawa R. (1995). Ligand-independent repression by the thyroid hormone receptor mediated by a nuclear receptor co-repressor. Nature 377 (6548), 397–404. 10.1038/377397a0 7566114

[B30] Huang S. Laoukili J. Epping M. T. Koster J. Hölzel M. Westerman B. A. (2009). ZNF423 is critically required for retinoic acid-induced differentiation and is a marker of neuroblastoma outcome. Cancer Cell 15 (4), 328–340. 10.1016/j.ccr.2009.02.023 19345331 PMC2693316

[B31] Janeckova L. Pospichalova V. Fafilek B. Vojtechova M. Tureckova J. Dobes J. (2015). HIC1 tumor suppressor loss potentiates TLR2/NF-κB signaling and promotes tissue damage-associated tumorigenesis. Mol. Cancer Res. 13 (7), 1139–1148. 10.1158/1541-7786.Mcr-15-0033 25934696

[B32] Janesick A. Wu S. C. Blumberg B. (2015). Retinoic acid signaling and neuronal differentiation. Cell. Mol. Life Sci. 72 (8), 1559–1576. 10.1007/s00018-014-1815-9 25558812 PMC11113123

[B33] Januchowski R. Wojtowicz K. Zabel M. (2013). The role of aldehyde dehydrogenase (ALDH) in cancer drug resistance. Biomed. Pharmacother. 67 (7), 669–680. 10.1016/j.biopha.2013.04.005 23721823

[B34] Jerónimo C. Henrique R. Oliveira J. Lobo F. Pais I. Teixeira M. R. (2004). Aberrant cellular retinol binding protein 1 (CRBP1) gene expression and promoter methylation in prostate cancer. J. Clin. Pathol. 57 (8), 872–876. 10.1136/jcp.2003.014555 15280411 PMC1770387

[B35] Kovalevich J. Langford D. (2013). Considerations for the use of SH-SY5Y neuroblastoma cells in neurobiology. Methods Mol. Biol. 1078, 9–21. 10.1007/978-1-62703-640-5_2 23975817 PMC5127451

[B36] Kuppumbatti Y. S. Bleiweiss I. J. Mandeli J. P. Waxman S. Mira-y-Lopez R. (2000). Cellular retinol-binding protein expression and breast cancer. J. Natl. Cancer Inst. 92 (6), 475–480. 10.1093/jnci/92.6.475 10716965

[B37] Lai A. Lee J. M. Yang W. M. DeCaprio J. A. Kaelin W. G. Jr. Seto E. (1999). RBP1 recruits both histone deacetylase-dependent and -independent repression activities to retinoblastoma family proteins. Mol. Cell. Biol. 19 (10), 6632–6641. 10.1128/mcb.19.10.6632 10490602 PMC84642

[B38] Langmead B. Salzberg S. L. (2012). Fast gapped-read alignment with Bowtie 2. Nat. Methods 9 (4), 357–359. 10.1038/nmeth.1923 22388286 PMC3322381

[B39] Lecca M. C. Jonker M. A. Abdul U. K. Küçükosmanoglu A. Wieringen W. v. Westerman B. A. (2018). Adrenergic to mesenchymal fate switching of neuroblastoma occurs spontaneously *in vivo* resulting in differential tumorigenic potential. JMCM 1 (4), 219–226. 10.31083/j.jmcm.2018.04.4221

[B40] Liu C. Lee M. K. Naqvi S. Hoskens H. Liu D. White J. D. (2021). Genome scans of facial features in East Africans and cross-population comparisons reveal novel associations. PLoS Genet. 17 (8), e1009695. 10.1371/journal.pgen.1009695 34411106 PMC8375984

[B41] Llères D. Moindrot B. Pathak R. Piras V. Matelot M. Pignard B. (2019). CTCF modulates allele-specific sub-TAD organization and imprinted gene activity at the mouse Dlk1-Dio3 and Igf2-H19 domains. Genome Biol. 20 (1), 272. 10.1186/s13059-019-1896-8 31831055 PMC6909504

[B42] Louis C. U. Shohet J. M. (2015). Neuroblastoma: Molecular pathogenesis and therapy. Annu. Rev. Med. 66, 49–63. 10.1146/annurev-med-011514-023121 25386934 PMC4418018

[B43] Love M. I. Huber W. Anders S. (2014). Moderated estimation of fold change and dispersion for RNA-seq data with DESeq2. Genome Biol. 15 (12), 550. 10.1186/s13059-014-0550-8 25516281 PMC4302049

[B44] Lovén J. Hoke H. A. Lin C. Y. Lau A. Orlando D. A. Vakoc C. R. (2013). Selective inhibition of tumor oncogenes by disruption of super-enhancers. Cell 153 (2), 320–334. 10.1016/j.cell.2013.03.036 23582323 PMC3760967

[B45] Maden M. (2007). Retinoic acid in the development, regeneration and maintenance of the nervous system. Nat. Rev. Neurosci. 8 (10), 755–765. 10.1038/nrn2212 17882253

[B46] Mansour M. R. Abraham B. J. Anders L. Berezovskaya A. Gutierrez A. Durbin A. D. (2014). Oncogene regulation. An oncogenic super-enhancer formed through somatic mutation of a noncoding intergenic element. Science 346 (6215), 1373–1377. 10.1126/science.1259037 25394790 PMC4720521

[B47] Masetti R. Biagi C. Zama D. Vendemini F. Martoni A. Morello W. (2012). Retinoids in pediatric onco-hematology: The model of acute promyelocytic leukemia and neuroblastoma. Adv. Ther. 29 (9), 747–762. 10.1007/s12325-012-0047-3 22941525

[B48] Matthay K. K. Maris J. M. Schleiermacher G. Nakagawara A. Mackall C. L. Diller L. (2016). Neuroblastoma. Nat. Rev. Dis. Prim. 2 (1), 16078. 10.1038/nrdp.2016.78 27830764

[B49] Matthay K. K. Villablanca J. G. Seeger R. C. Stram D. O. Harris R. E. Ramsay N. K. (1999). Treatment of high-risk neuroblastoma with intensive chemotherapy, radiotherapy, autologous bone marrow transplantation, and 13-cis-retinoic acid. Children's Cancer Group. N. Engl. J. Med. 341 (16), 1165–1173. 10.1056/nejm199910143411601 10519894

[B50] Meyers E. A. Kessler J. A. (2017). TGF-Β family signaling in neural and neuronal differentiation, development, and function. Cold Spring Harb. Perspect. Biol. 9 (8), a022244. 10.1101/cshperspect.a022244 28130363 PMC5538418

[B51] Millet C. Zhang Y. E. (2007). Roles of Smad3 in TGF-beta signaling during carcinogenesis. Crit. Rev. Eukaryot. Gene Expr. 17 (4), 281–293. 10.1615/critreveukargeneexpr.v17.i4.30 17725494 PMC2639747

[B52] Napoli J. L. (2012). Physiological insights into all-trans-retinoic acid biosynthesis. Biochim. Biophys. Acta 1821 (1), 152–167. 10.1016/j.bbalip.2011.05.004 21621639 PMC3179567

[B53] Nilson K. A. Guo J. Turek M. E. Brogie J. E. Delaney E. Luse D. S. (2015). THZ1 reveals roles for Cdk7 in Co-transcriptional capping and pausing. Mol. Cell 59 (4), 576–587. 10.1016/j.molcel.2015.06.032 26257281 PMC4546572

[B54] Oppenheimer O. Cheung N.-K. Gerald W. L. (2007). The RET oncogene is a critical component of transcriptional programs associated with retinoic acid–induced differentiation in neuroblastoma. Mol. Cancer Ther. 6 (4), 1300–1309. 10.1158/1535-7163.Mct-06-0587 17431108

[B55] Otte J. Dyberg C. Pepich A. Johnsen J. I. (2021). MYCN function in neuroblastoma development. Front. Oncol. 10, 624079. 10.3389/fonc.2020.624079 33585251 PMC7873735

[B56] Pasini D. Cloos P. A. Walfridsson J. Olsson L. Bukowski J. P. Johansen J. V. (2010). JARID2 regulates binding of the Polycomb repressive complex 2 to target genes in ES cells. Nature 464 (7286), 306–310. 10.1038/nature08788 20075857

[B57] Rathi A. Virmani A. K. Harada K. Timmons C. F. Miyajima K. Hay R. J. (2003). Aberrant methylation of the HIC1 promoter is a frequent event in specific pediatric neoplasms. Clin. Cancer Res. 9 (10), 3674–3678. 14506157

[B58] Ray H. Chang C. (2020). The transcription factor Hypermethylated in Cancer 1 (Hic1) regulates neural crest migration via interaction with Wnt signaling. Dev. Biol. 463 (2), 169–181. 10.1016/j.ydbio.2020.05.012 32502469 PMC7437249

[B59] Ren M. Pozzi S. Bistulfi G. Somenzi G. Rossetti S. Sacchi N. (2005). Impaired retinoic acid (RA) signal leads to RARbeta2 epigenetic silencing and RA resistance. Mol. Cell. Biol. 25 (23), 10591–10603. 10.1128/MCB.25.23.10591-10603.2005 16287870 PMC1291229

[B60] Rettig I. Koeneke E. Trippel F. Mueller W. C. Burhenne J. Kopp-Schneider A. (2015). Selective inhibition of HDAC8 decreases neuroblastoma growth *in vitro* and *in vivo* and enhances retinoic acid-mediated differentiation. Cell Death Dis. 6 (2), e1657. 10.1038/cddis.2015.24 25695609 PMC4669789

[B61] Reynolds C. P. Matthay K. K. Villablanca J. G. Maurer B. J. (2003). Retinoid therapy of high-risk neuroblastoma. Cancer Lett. 197 (1-2), 185–192. 10.1016/s0304-3835(03)00108-3 12880980

[B62] Ross-Innes C. S. Stark R. Teschendorff A. E. Holmes K. A. Ali H. R. Dunning M. J. (2012). Differential oestrogen receptor binding is associated with clinical outcome in breast cancer. Nature 481 (7381), 389–393. 10.1038/nature10730 22217937 PMC3272464

[B63] Saint-André V. Federation A. J. Lin C. Y. Abraham B. J. Reddy J. Lee T. I. (2016). Models of human core transcriptional regulatory circuitries. Genome Res. 26 (3), 385–396. 10.1101/gr.197590.115 26843070 PMC4772020

[B64] Sengupta S. George R. E. (2017). Super-enhancer-driven transcriptional dependencies in cancer. Trends Cancer 3 (4), 269–281. 10.1016/j.trecan.2017.03.006 28718439 PMC5546010

[B65] Shipley M. M. Mangold C. A. Szpara M. L. (2016). Differentiation of the SH-SY5Y human neuroblastoma cell line. J. Vis. Exp. (108), 53193. 10.3791/53193 26967710 PMC4828168

[B66] Sidell N. Altman A. Haussler M. R. Seeger R. C. (1983). Effects of retinoic acid (RA) on the growth and phenotypic expression of several human neuroblastoma cell lines. Exp. Cell Res. 148 (1), 21–30. 10.1016/0014-4827(83)90184-2 6313408

[B67] Smith V. Foster J. (2018). High-risk neuroblastoma treatment review. Child. (Basel, Switz. 5 (9), 114. 10.3390/children5090114 PMC6162495 30154341

[B68] Southgate H. E. D. Chen L. Curtin N. J. Tweddle D. A. (2020). Targeting the DNA damage response for the treatment of high risk neuroblastoma. Front. Oncol. 10, 371. 10.3389/fonc.2020.00371 32309213 PMC7145987

[B69] Stark R. Brown G. (2012). DiffBind: Differential binding analysis of ChIP-Seq peak data. Cambridge: Bioconductor.

[B70] Toki K. Enokida H. Kawakami K. Chiyomaru T. Tatarano S. Yoshino H. (2010). CpG hypermethylation of cellular retinol-binding protein 1 contributes to cell proliferation and migration in bladder cancer. Int. J. Oncol. 37 (6), 1379–1388. 10.3892/ijo_00000789 21042705

[B71] Tomolonis J. A. Agarwal S. Shohet J. M. (2018). Neuroblastoma pathogenesis: Deregulation of embryonic neural crest development. Cell Tissue Res. 372 (2), 245–262. 10.1007/s00441-017-2747-0 29222693 PMC5918240

[B72] Urvalek A. M. Gudas L. J. (2014). Retinoic acid and histone deacetylases regulate epigenetic changes in embryonic stem cells. J. Biol. Chem. 289 (28), 19519–19530. 10.1074/jbc.M114.556555 24821725 PMC4094062

[B73] van Groningen T. Akogul N. Westerhout E. M. Chan A. Hasselt N. E. Zwijnenburg D. A. (2019). A NOTCH feed-forward loop drives reprogramming from adrenergic to mesenchymal state in neuroblastoma. Nat. Commun. 10 (1), 1530. 10.1038/s41467-019-09470-w 30948783 PMC6449373

[B74] van Groningen T. Koster J. Valentijn L. J. Zwijnenburg D. A. Akogul N. Hasselt N. E. (2017). Neuroblastoma is composed of two super-enhancer-associated differentiation states. Nat. Genet. 49, 1261–1266. 10.1038/ng.3899 28650485

[B75] van Groningen T. Niklasson C. U. Chan A. Akogul N. Westerhout E. M. von Stedingk K. (2021). An immature subset of neuroblastoma cells synthesizes retinoic acid and depends on this metabolite. bioRxiv. 10.1101/2021.05.18.444639

[B76] Wan H. Oridate N. Lotan D. Hong W. K. Lotan R. (1999). Overexpression of retinoic acid receptor β in head and neck squamous cell carcinoma cells increases their sensitivity to retinoid-induced suppression of squamous differentiation by retinoids. Cancer Res. 59 (14), 3518–3526. 10416619

[B77] Wang X. Cairns M. J. Yan J. (2019). Super-enhancers in transcriptional regulation and genome organization. Nucleic Acids Res. 47 (22), 11481–11496. 10.1093/nar/gkz1038 31724731 PMC7145697

[B78] Westerhout E. M. Hamdi M. Stroeken P. Nowakowska N. E. Lakeman A. van Arkel J. (2022). Mesenchymal-type neuroblastoma cells escape ALK inhibitors. Cancer Res. 82 (3), 484–496. 10.1158/0008-5472.Can-21-1621 34853072

[B79] Westerlund I. Shi Y. Toskas K. Fell S. M. Li S. Surova O. (2017). Combined epigenetic and differentiation-based treatment inhibits neuroblastoma tumor growth and links HIF2α to tumor suppression. Proc. Natl. Acad. Sci. U. S. A. 114 (30), E6137–E6146. 10.1073/pnas.1700655114 28696319 PMC5544284

[B80] Whyte W. A. Orlando D. A. Hnisz D. Abraham B. J. Lin C. Y. Kagey M. H. (2013). Master transcription factors and mediator establish super-enhancers at key cell identity genes. Cell 153 (2), 307–319. 10.1016/j.cell.2013.03.035 23582322 PMC3653129

[B81] Wimmer K. Zhu Xx X. X. Rouillard J. M. Ambros P. F. Lamb B. J. Kuick R. (2002). Combined restriction landmark genomic scanning and virtual genome scans identify a novel human homeobox gene, ALX3, that is hypermethylated in neuroblastoma. Genes Chromosom. Cancer 33 (3), 285–294. 10.1002/gcc.10030 11807986

[B82] Yan X. Kennedy C. R. Tilkens S. B. Wiedemeier O. Guan H. Park J. I. (2011). Cooperative cross-talk between neuroblastoma subtypes confers resistance to anaplastic lymphoma kinase inhibition. Genes Cancer 2 (5), 538–549. 10.1177/1947601911416003 21901167 PMC3161418

[B83] Zeid R. Lawlor M. A. Poon E. Reyes J. M. Fulciniti M. Lopez M. A. (2018). Enhancer invasion shapes MYCN-dependent transcriptional amplification in neuroblastoma. Nat. Genet. 50 (4), 515–523. 10.1038/s41588-018-0044-9 29379199 PMC6310397

[B84] Zhang G. Feenstra B. Bacelis J. Liu X. Muglia L. M. Juodakis J. (2017). Genetic associations with gestational duration and spontaneous preterm birth. N. Engl. J. Med. 377 (12), 1156–1167. 10.1056/NEJMoa1612665 28877031 PMC5561422

[B85] Zhang W. Zeng X. Briggs K. J. Beaty R. Simons B. Chiu Yen R. W. (2010). A potential tumor suppressor role for Hic1 in breast cancer through transcriptional repression of ephrin-A1. Oncogene 29 (17), 2467–2476. 10.1038/onc.2010.12 20154726 PMC3025282

[B86] Zhang Y. Liu T. Meyer C. A. Eeckhoute J. Johnson D. S. Bernstein B. E. (2008). Model-based analysis of ChIP-seq (MACS). Genome Biol. 9 (9), R137. 10.1186/gb-2008-9-9-r137 18798982 PMC2592715

[B87] Zimmerman M. W. Durbin A. D. He S. Oppel F. Shi H. Tao T. (2021). Retinoic acid rewires the adrenergic core regulatory circuitry of childhood neuroblastoma. Sci. Adv. 7 (43), eabe0834. 10.1126/sciadv.abe0834 34669465 PMC8528416

