# Supplementary material for: Super-enhancer associated core regulatory circuits mediate susceptibility to retinoic acid in neuroblastoma cells
Source: Front Cell Dev Biol. 2022 Sep 6;10:943924. doi: 10.3389/fcell.2022.943924 (PMC9485839; doi:10.3389/fcell.2022.943924)
Supplement: Supplementary file 1 [file DataSheet1.pdf]

Supplementary Material

Supplementary Movie S1: Time-lapse movie showing BE2C cells maintaining the differentiated phenotype after withdrawal of ATRA.

Supplementary Movie S2: Time-lapse movie showing SH-SY5Y cells reverting after withdrawal of ATRA

Supplementary Figure S1

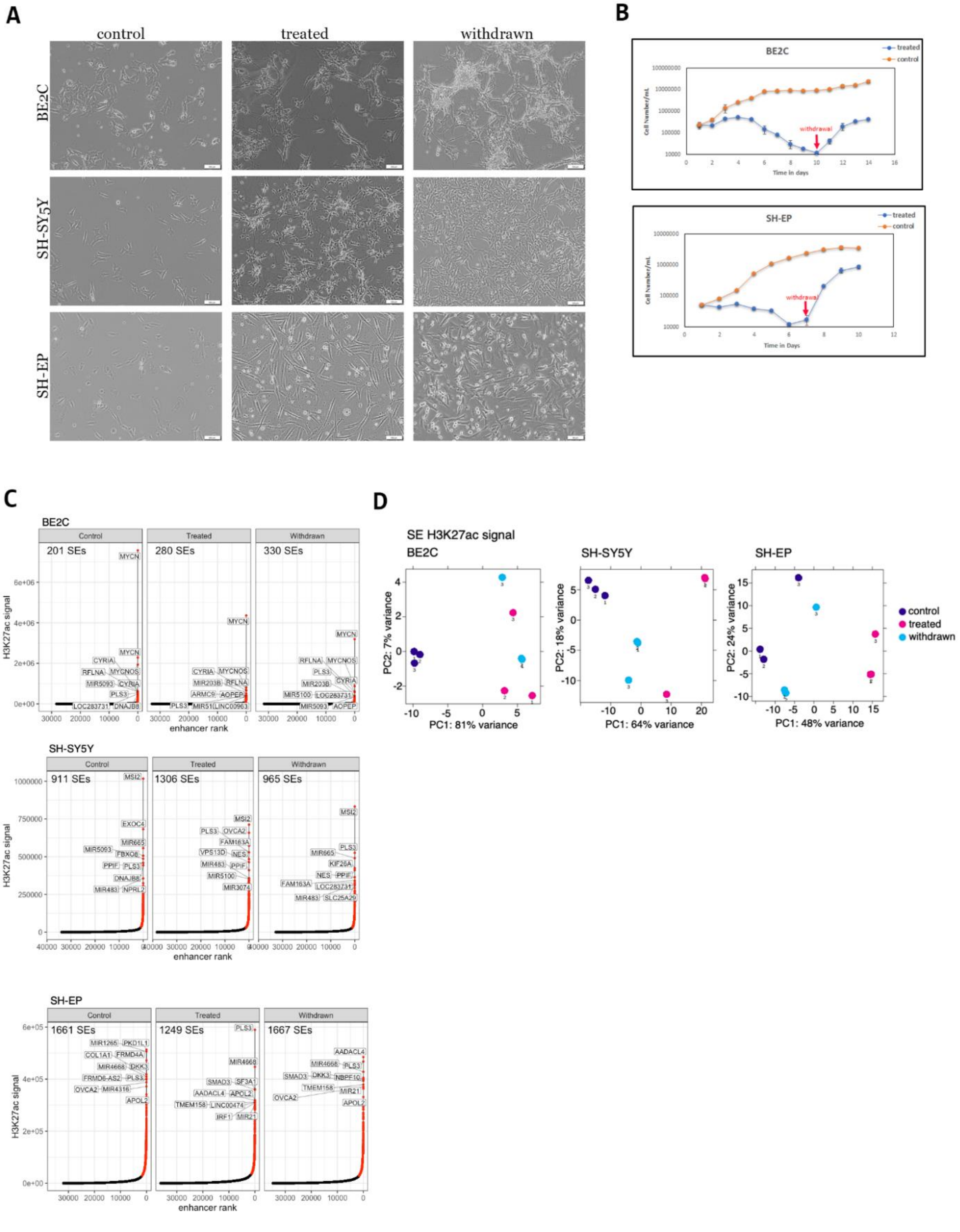

Supplementary Figure S2

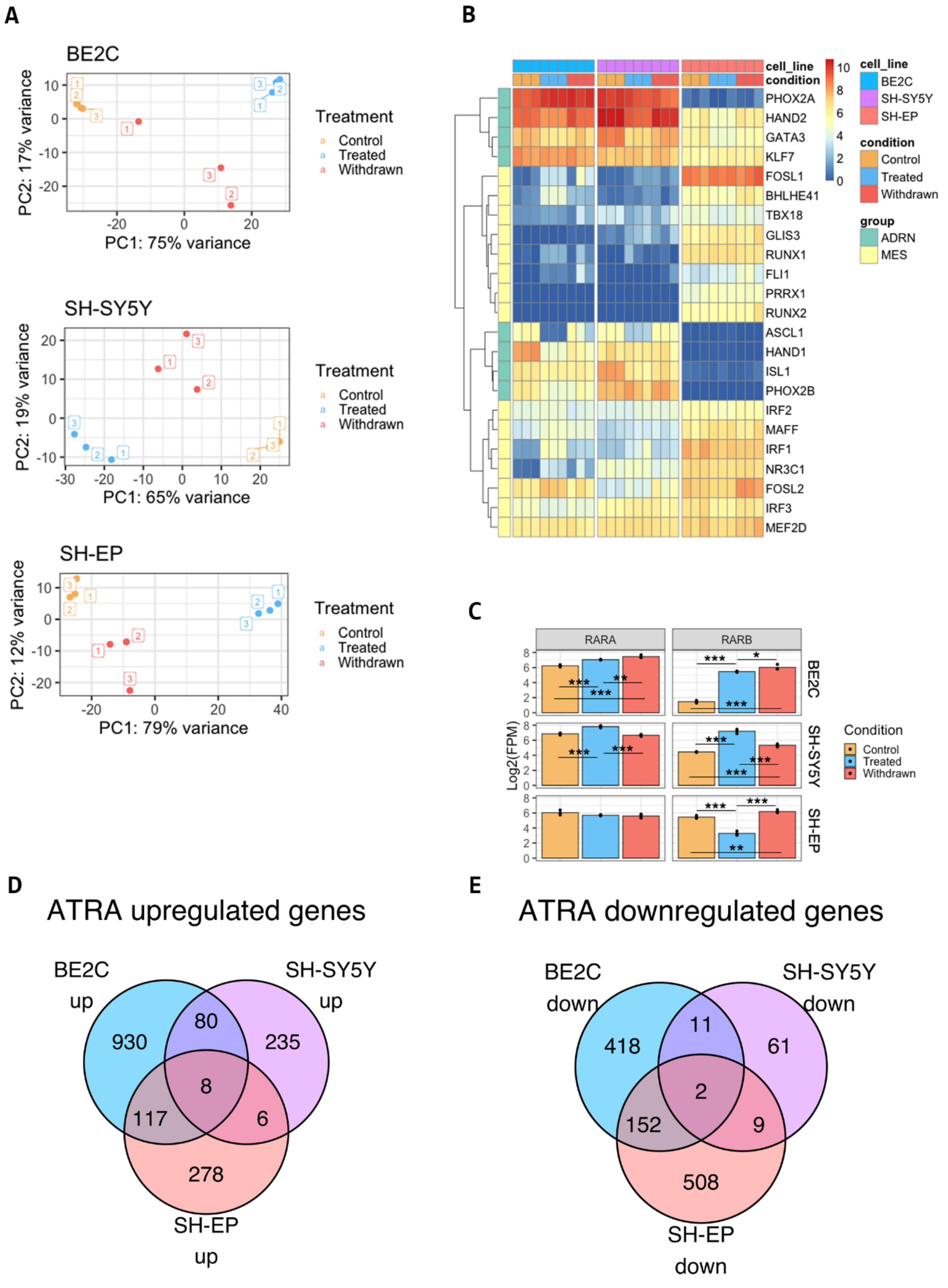

Supplementary Figure S3

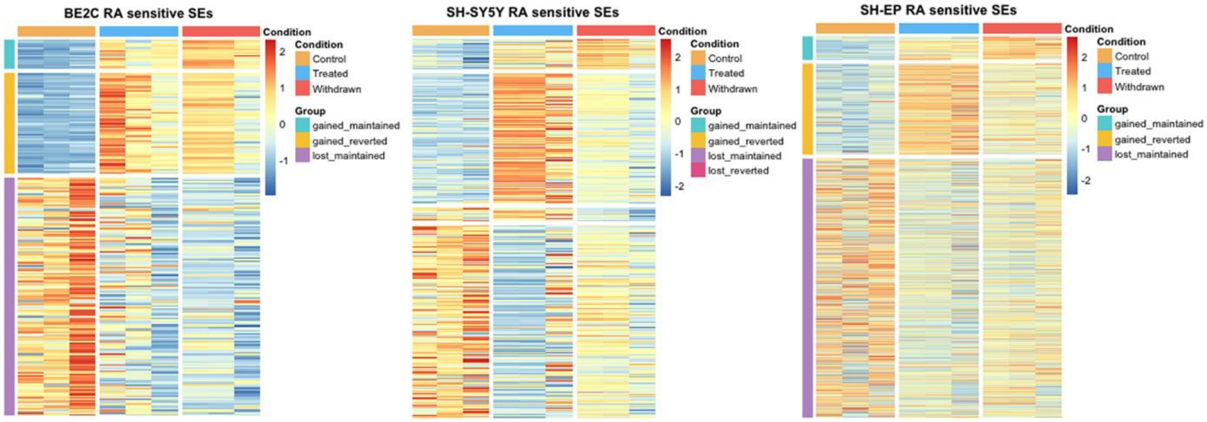

Supplementary Figure S4

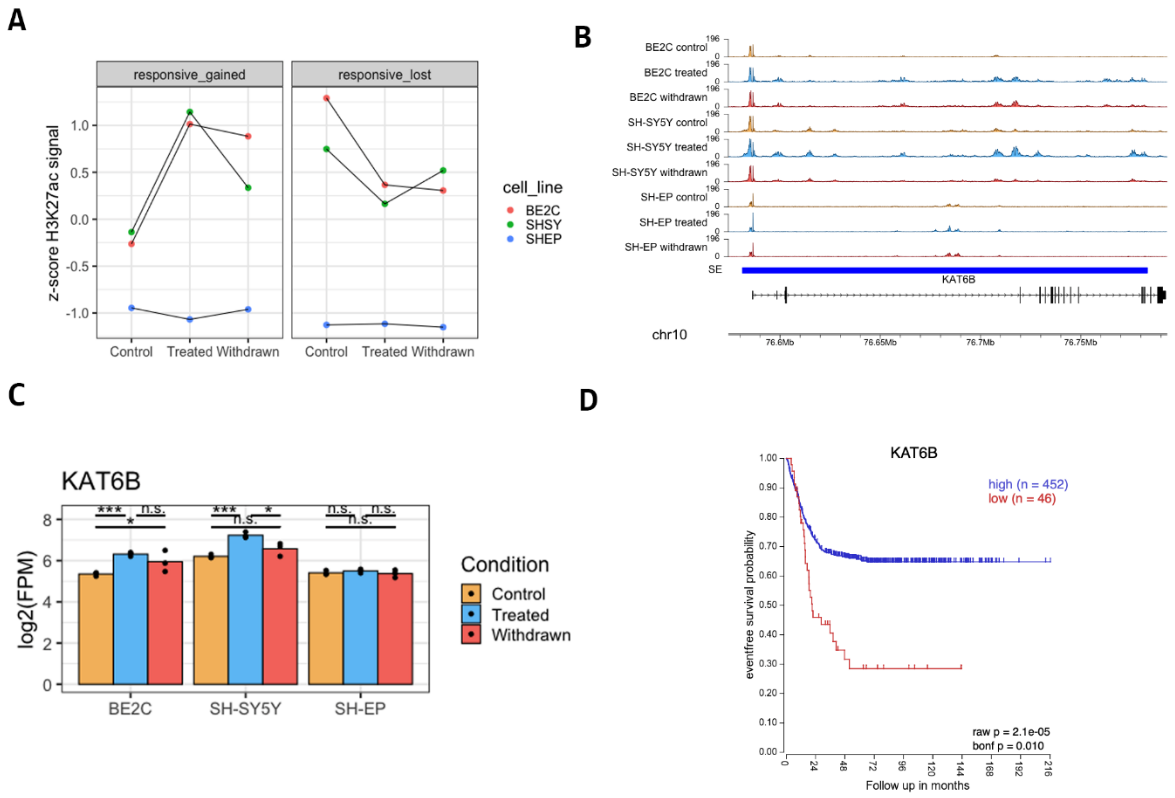

Supplementary figure S5

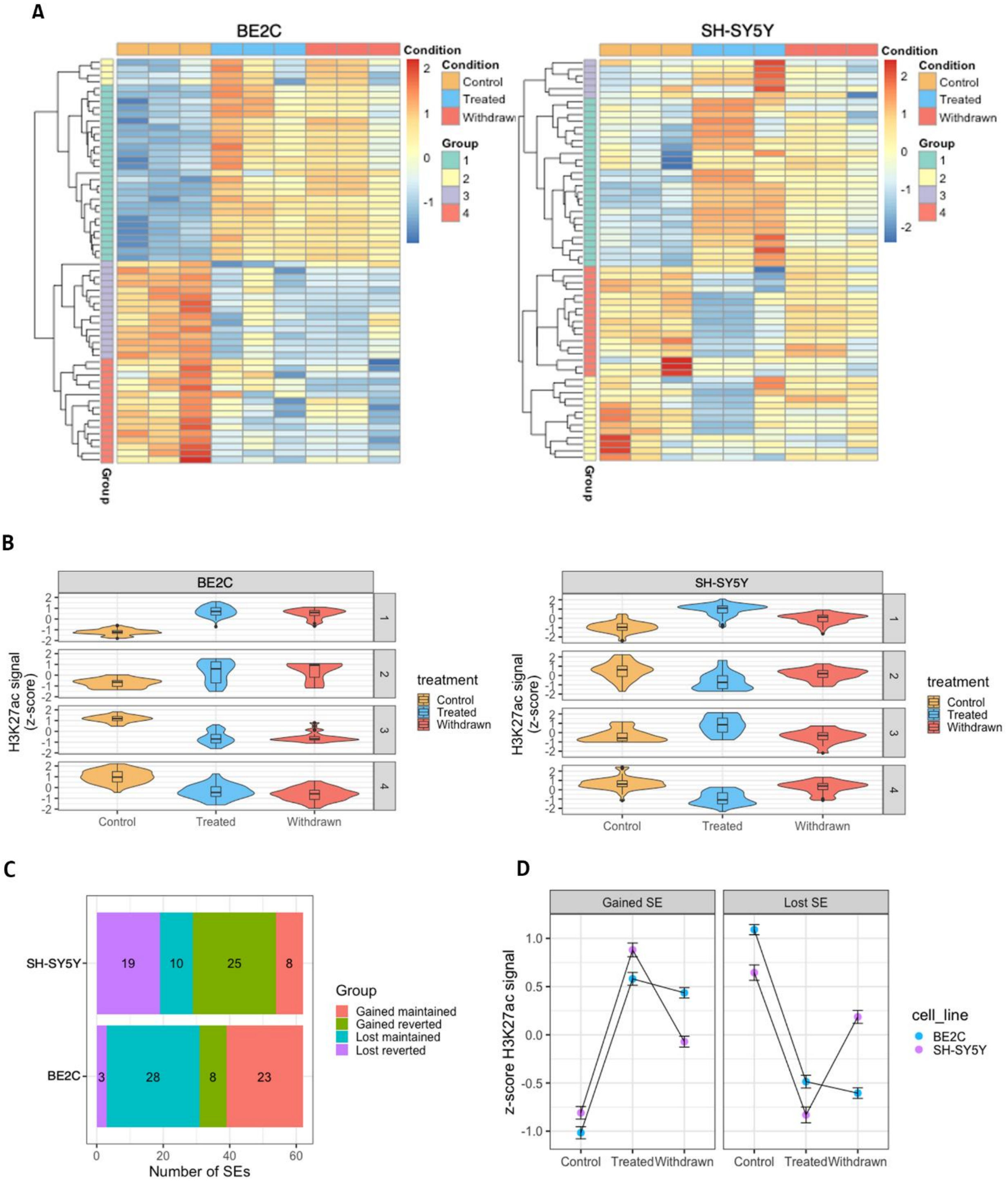

Supplementary Figure S6

A

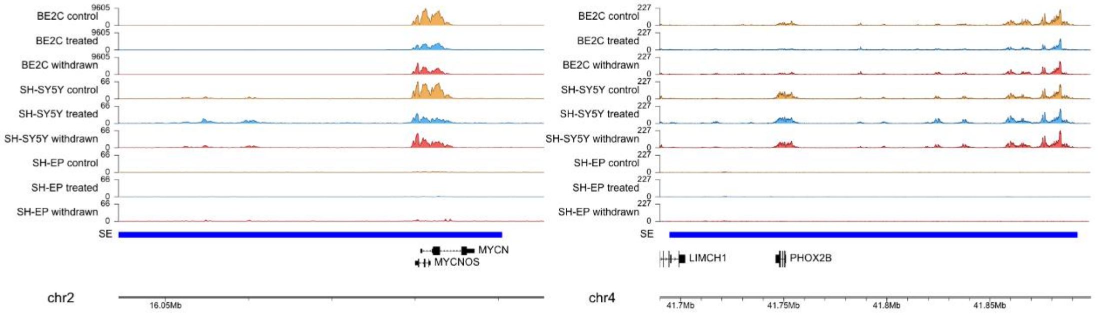

B

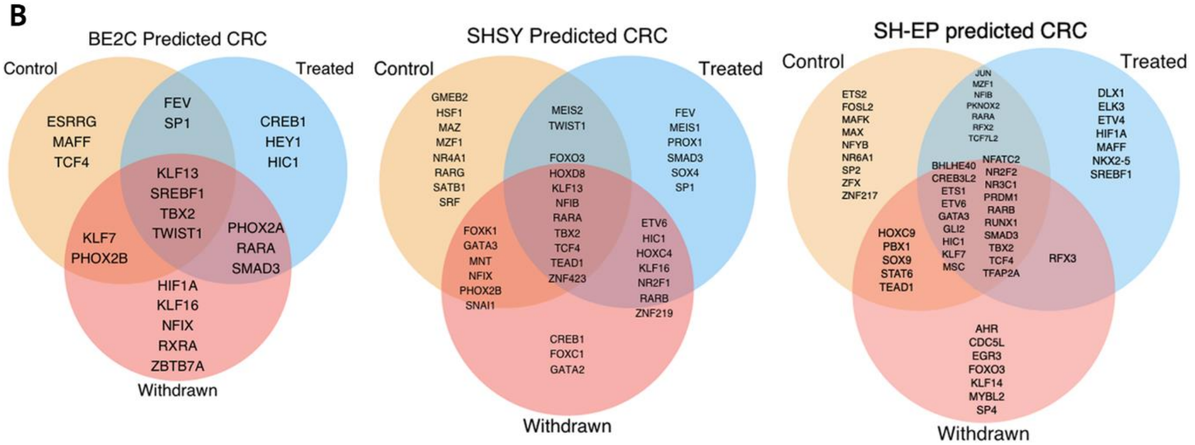

C

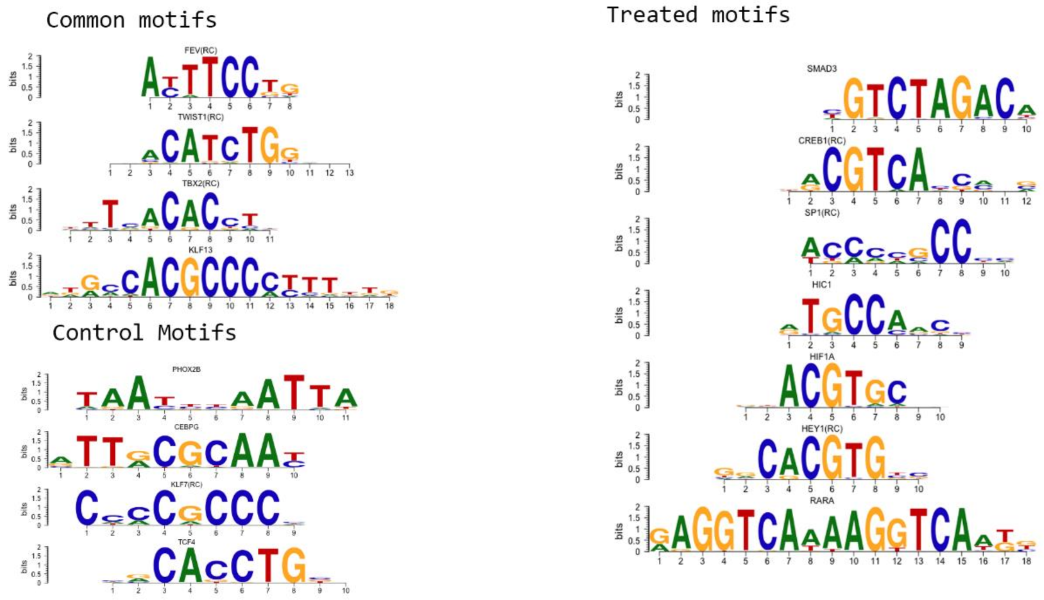

Supplementary Figure S7

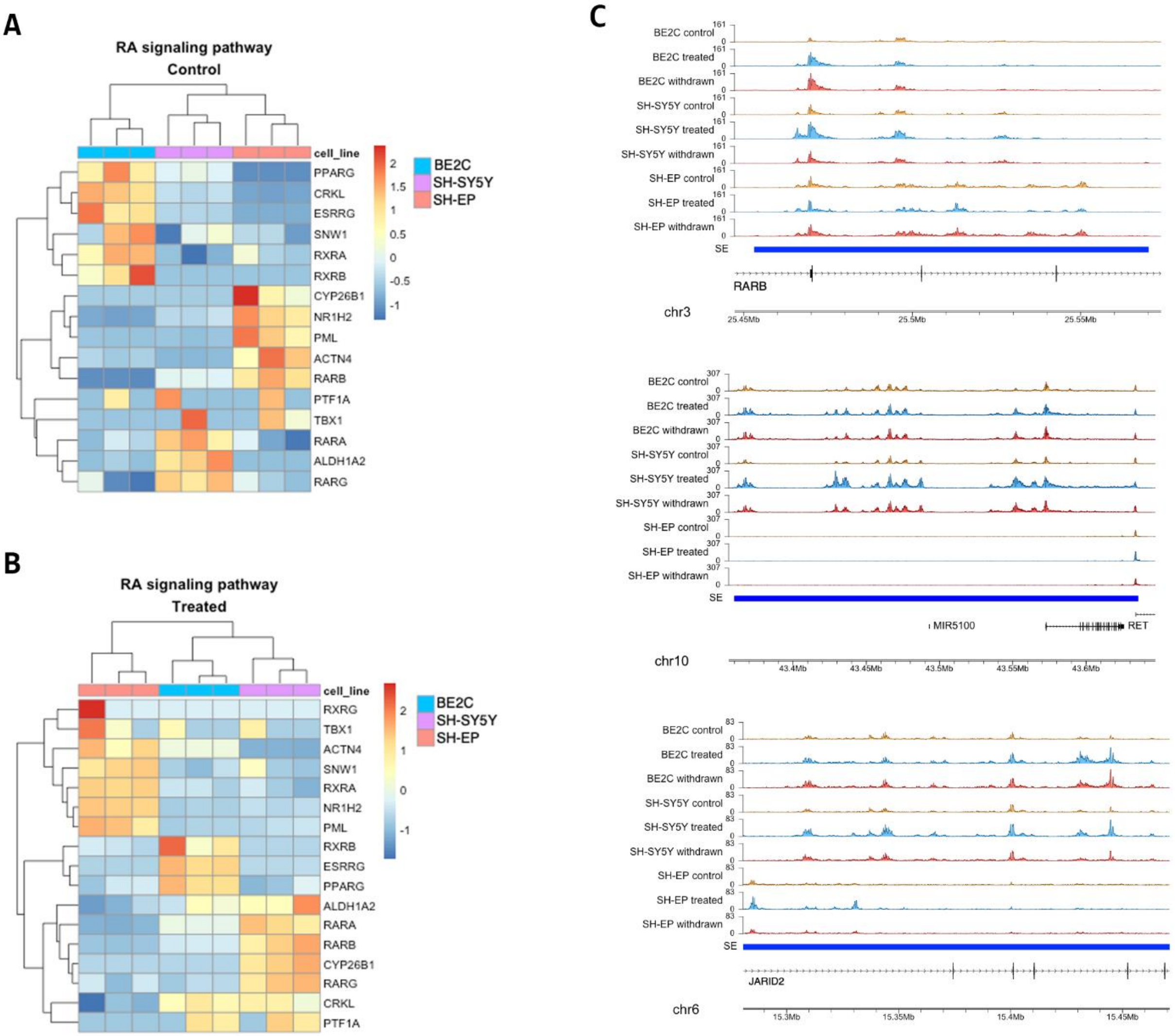

Supplementary Figure Legends

Supplementary figure S1: (A) Phase contrast microscopy images of responsive cell lines (BE2C, SH-SY5Y) and resistant cell line (SH-EP) under control, upon ATRA treatment and subsequent removal of ATRA. Scale bar indicates 100 µM (except for 'SH-EP withdrawn' which is 50 µM). **(B)** Growth curve of responsive (BE2C) and resistant (SH-EP) cell lines grown in 24-well plate. Each value is the average of cell counts from 2 wells, taken at an interval of 24 hours. Time point of withdrawal is marked in red arrow. **(C)** Ranked enhancer plots, showing H3K27ac signal at enhancers and super-enhancers in BE2C, SH-SY5Y and SH-EP under each condition identified using ROSE. Regions designated as super-enhancers highlighted in red, the top 10 super-enhancers from each condition are annotated with their predicted target gene. **(D)** PCA of H3K27ac signal at super-enhancer regions identified by ROSE in control, treated, or withdrawn conditions for each cell line.

Supplementary figure S2: (A) PCA of RNA-seq data in each condition, for each cell line. **(B)** Heat map showing expression of ADRN and MES markers in the 3 different cell lines after ATRA treatment and withdrawal. **(C)** Bar plots showing the expression of *RARA* and *RARB* in control, treated and withdrawn conditions in responsive cell lines (BE2C and SH-SY5Y) and resistant cell line (SH-EP). Adjusted p-value displayed; Statistical significance determined using DESeq2 (*padj<0.05, **padj<0.01, ***padj<0.001). Venn diagram shows genes significantly upregulated **(D)** and downregulated **(E)** after ATRA treatment in each cell line (padj < 0.05, log2FC > 2).

Supplementary figure S3: Heatmap showing normalised H3K27ac signal (z-score) at ATRA responsive super-enhancers identified in BE2C, SH-SY5Y and SH-EP, and grouped by patterns of H3K27ac change.

Supplementary figure S4: (A) Average change in H3K27ac signal at gained or lost super-enhancers in responsive (BE2C and SH-SY5Y) and resistant cell lines (SH-EP). Y-axis shows z-score average expression for each group of super-enhancers. **(B)** Representative ChIP-seq tracks displaying super-enhancer regions of *KAT6B* identified in ATRA responsive cell lines. **(C)** Bar plot showing the expression of *KAT6B* in control, treated and withdrawn conditions in responsive cell lines (BE2C and SH-SY5Y) and resistant cell line (SH-EP). Statistical significance determined using DESeq2 (*padj<0.05, **padj<0.01, ***padj<0.001). **(D)** Kaplan-Meier survival curves showing the probability of event free survival in two groups of neuroblastoma patients, split based on their tumor expression level of *KAT6B*.

Supplementary figure S5: (A) Heatmap showing changes in H3K27ac levels at super-enhancers which gain or lose H3K27ac in response to ATRA treatment in BE2C and SH-SY5Y cell lines. Data is z-scaled for each super-enhancer region, within each cell line. Data was split into 4 groups for each cell line by hierarchical clustering, based on the pattern of H3K27ac after ATRA treatment and withdrawal **(B)** Violin plots summarizing the H3K27ac signal data shown in Supplementary Figure 5A. **(C)** Bar chart showing the number of super-enhancers in each of the four groups identified in the violin plot in each cell line (SH-SY5Y and BE2C). **(D)** Average change in H3K27ac

signal at gained or lost super-enhancers shown in Figure S5A in BE2C and SH-SY5Y. Y-axis shows z-score H3K27ac for each group of SEs.

Supplementary figure S6: (A) Representative ChIP-seq tracks displaying super-enhancers associated with *MYCN* (left panel) and *PHOX2B* (right panel). **(B)** Venn diagram showing the predicted CRC TFs of each cell line under control, treated and ATRA withdrawn conditions. **(C)** Binding motifs of the predicted CRCs unique to control and treated conditions and those which are common to both conditions.

Supplementary figure S7: Heatmaps showing the change in expression of retinoic acid signaling pathway genes in **(A)** control and **(B)** treated conditions, z-scaled by row. **(C)** Representative ChIP-seq tracks displaying super-enhancers associated with RARB (upper panel), RET (middle panel) and JARID2 (lower panel).
